# Immunohistochemical analysis of PDK1, PHD3 and HIF-1α expression defines the hypoxic status of neuroblastoma tumors

**DOI:** 10.1371/journal.pone.0187206

**Published:** 2017-11-08

**Authors:** Marzia Ognibene, Davide Cangelosi, Martina Morini, Daniela Segalerba, Maria Carla Bosco, Angela Rita Sementa, Alessandra Eva, Luigi Varesio

**Affiliations:** 1 Laboratory of Molecular Biology, Giannina Gaslini Institute, Genova, Italy; 2 Department of Pathology, Giannina Gaslini Institute, Genova, Italy; University of South Alabama Mitchell Cancer Institute, UNITED STATES

## Abstract

Neuroblastoma (NB) is the most common solid tumor during infancy and the first cause of death among the preschool age diseases. The availability of several NB genomic profiles improves the prognostic ability, but the outcome prediction for this pathology remains imperfect. We previously produced a novel prognostic gene signature based on the response of NB cells to hypoxia, a condition of tumor microenvironment strictly connected with cancer aggressiveness. Here we attempted to further define the expression of hypoxia-modulated specific genes, looking at their protein level in NB specimens, considering in particular the hypoxia inducible factor-1α (HIF-1α), the mitochondrial pyruvate dehydrogenase kinase 1 (PDK1), and the HIF-prolyl hydroxylase domain 3 (PHD3). The evaluation of expression was performed by Western blot and immunocytochemistry on NB cell lines and by immunohistochemistry on tumor specimens. Stimulation of both HIF-1α and PDK1 and inhibition of PHD3 expression were observed in NB cell lines cultured under prolonged hypoxic conditions as well as in most of the tumors with poor outcome. Our results indicate that the immunohistochemistry analysis of the protein expression of PDK1, PHD3, and HIF-1α defines the hypoxic status of NB tumors and can be used as a simple and relevant tool to stratify high-risk patients.

## Introduction

Neuroblastoma (NB) is the most common solid tumor in childhood and originates in the neural crest from ganglionic lineage precursors of the sympathetic nervous system [1,2]. NB accounts for more than 7% of malignancies in patients younger than 15 years and causes 15% of all pediatric oncology deaths [3,4]. NB shows a remarkable heterogeneity with regard to histology and clinical behavior [5], ranging from rapid progression associated with metastatic spread and poor clinical outcome to spontaneous or therapy-induced regression into benign ganglioneuroma [6]. Clinical and molecular risk factors that correlate with prognosis include age at diagnosis, stage, histology, chromosomal aberrations, and amplification of the N-Myc proto-oncogene (*MYCN*), which is the most typical genetic feature of advanced-stage NB [5,7,8]. In fact, *MYCN* amplification correlates with a more malignant course of the disease, angiogenesis, resistance to therapy, and poor clinical outcome [7,9–11], suggesting that it may be a progression-related event and a potential therapeutic target [5].

The availability of NB genomic profiles has improved the prognostic ability. Several groups have developed gene expression-based approaches to stratify NB patients [12–18] and described prognostic gene signatures that were more robust than single biomarkers because they are based on the concomitant assessment of multiple genes [19]. Nevertheless, despite elaborate risk estimation strategies, outcome prediction for patients with NB is still imperfect.

We studied outcome prediction in NB patients utilizing a biology-driven approach in which the gene expression profile under investigation is associated with the a priori knowledge on a biological process that impacts on tumor growth [20]. Specifically, we studied the response of NB cells to hypoxia and used this information to derive a novel prognostic gene signature [21].

Tumor microenvironment is intimately connected with the evolution of the disease. In particular, hypoxia, a condition of low oxygen tension occurring in poorly vascularized areas, has a profound effect on tumor cell growth, genotype selection, susceptibility to apoptosis, resistance to radio- and chemotherapy, tumor angiogenesis, epithelial to mesenchymal transition, and propagation of cancer stem cells [22–24]. There is little information on the relationship among hypoxia, tumor phenotypes, and clinical parameters in NB. Rapidly expanding NB tumors present hypoxic areas and metastasize to bone marrow [25]. Adaptation of NB cells to hypoxia activates a gene expression program consistent with pro-metastatic events [26]. Furthermore, hypoxia causes dedifferentiation *in vitro* and *in vivo*, suggesting a novel mechanism for the selection of highly malignant NB cells with stem-cell characteristics [27].

In our previous work, we investigated the prognostic potential of hypoxia-induced genes in NB tumors by performing a systematic analysis of the transcriptome of NB cell lines cultured under hypoxic or normoxic conditions and derived a robust 62 probesets NB hypoxia signature (NB-hypo) [21]. We demonstrated that NB-hypo is an independent prognostic factor for NB, supporting the view that hypoxia is negatively correlated with patients' outcome [15,18]. Moreover, we generated rules classifying NB patients on the basis of NB-hypo and clinical and molecular risk factors and demonstrated that NB-hypo could be successfully associated to other known risk factors to generate relevant prognostic rules capable to stratify high risk patients [28]. The definition of hypoxia signatures for prognostic applications raises the question about their relationship with the type and degree of tumor hypoxia that can be independently assessed in the tumor samples, i.e. whether the gene products derived from the gene signatures can be used to monitor tumor hypoxia.

In the present study we attempted to further define the hypoxia-induced modulation of expression of specific genes in NB tumors. Analysis of the hypoxia markers related to the oxygen concentration was performed at the protein level on three NB cell lines and then tested on 25 tumor specimens previously classified as high or low risk on the basis of NB-hypo. We analyzed in these cell lines the protein expression of hypoxia inducible factor-1α (HIF-1α), which functions as a master regulator of oxygen homeostasis in all metazoan species [29], of glycolytic 6-phosphofructo-2-kinase (PFKFB4), one of the enzymes involved in the anaerobic glycolysis [30], of mitochondrial pyruvate dehydrogenase kinase 1 (PDK1), the enzyme that is critical for the attenuation of mitochondrial ROS production, maintenance of ATP levels, and adaptation to hypoxia [31], of vascular endothelial growth factor A (VEGFA), the signal protein that stimulates the formation of new blood vessels [32], and of HIF-prolyl hydroxylase domain 3 (PHD3), the enzyme that hydroxylates HIF-1α leading to limited steady-state levels of HIF-α under hypoxic conditions and to accelerated degradation after reoxygenation [33]. We show that culture under hypoxic conditions modulates the protein expression of these genes in NB cells. Furthermore, we demonstrate that the expression of PDK1, PHD3, and HIF-1α proteins in NB tumor samples can be used in immunohistochemistry to evaluate the hypoxic status of NB tumors, and could, therefore, be successfully used as a relevant tool to stratify high-risk patients.

## Materials and methods

### Patients

All tumor NB specimens were obtained from the BIT-Gaslini biobank that is an independent unit of the G. Gaslini Institute approved by the local regional ethical committee. Tissues samples were banked in the BIT-Gaslini biobank upon acceptance of the informed consent. The BIT-Gaslini biobank serves research functions. The samples are anonymized and the personal data are not disclosed to any researcher.

The samples analyzed in the present study were all peripheral neuroblastic tumors. This term defines a family of tumors, which derive from neuroectodermal embryonic cells and which include entities with different cellular composition and degrees of differentiation.

The cases have been classified according to the International Neuroblastoma Pathological Classification (INPC) [34], which distinguishes between different categories of Neuroblastic tumors and defines the prognostic impact of each category. For neuroblastoma, age and two microscopic features, mitosis karyorrhexis index (MKI) and grade of differentiation, have to be considered. DNA content was analyzed by means of cytofluorimetric assay on FFPE samples. Paraffin-embedded tissue sections of 4 μm were digested in 1% pepsin, and cell suspensions were stained in a solution of propidium iodine (50 mg/l), Triton X-100 (1 ml/l) and RNAse (75 KU/ml). Cytofluorimetric examination was carried out under a cytofluorimeter (FACScan Becton Dickinson, Franklin Lakes, NJ, USA) on at least 20.000 events. The results were analyzed by means of Modfit software [35].

For the majority of cases, a sample mirroring the one used for bio-molecular characterization was available for histological examination. Age at the diagnosis is also recorded. Whenever these parameters were available, the INPC prognostic grouping was applied and cases stratified as Favorable Histology (FH) and Unfavorable Histology (UH), according to the Shimada classification [34].

Tumor stage was defined according to the International NB Staging System (INSS) [36].

The clinical characteristics of the 25 NB tumors used in this study are detailed in Table 1.

**Table 1:** List of the 25 NB pediatric patients analyzed.

TUMOR NUMBER	CLUSTER	SEX	AGE AT DIAGNOSIS (months)	STAGE (INSS)	ONSET SITE	OUTCOME^1^	DNA PLOIDY^2^	HISTOLOGY^3^	N-myc^4^	HIF-1α^5^	PDK1^5^	PHD3^5^
1	**Hypoxic**	male	16	4	Abdomen-adrenal gland	Relapsed	1	UH	-	71–100%	71–100%	1–20%
2	**Hypoxic**	male	17	4	Thorax-abdomen	Deceased	2.03	UH	+	71–100%	71–100%	1–20%
3	**Hypoxic**	male	43	4	Abdomen-adrenal gland	Deceased	1	UH	+	71–100%	71–100%	1–20%
4	**Hypoxic**	male	55	4	Abdomen-adrenal gland	Deceased	1	UH	-	71–100%	51–70%	1–20%
5	**Hypoxic**	male	9	4S	Abdomen-retroperitoneal ganglia	Resid. disease	1	FH	+	71–100%	71–100%	1–20%
6	**Hypoxic**	female	21	3	Abdomen	Deceased	na	UH	+	71–100%	51–70%	<1%
7	**Hypoxic**	male	40	3	Abdomen-adrenal gland	Relapsed	2.01	UH	-	51–70%	21–50%	<1%
8	**Hypoxic**	female	69	1	Abdomen	Cont. remiss.	na	FH	+	71–100%	51–70%	1–20%
												
9	**Normoxic**	male	0.71	4	Abdomen-retroperitoneal ganglia	Resid. disease	na	FH	+	1–20%	1–20%	1–20%
10	**Normoxic**	male	7	4S	Abdomen-retroperitoneal ganglia	Resid. disease	1.74	FH	-	1–20%	1–20%	51–70%
11	**Normoxic**	male	5	3	Abdomen-pancreas	Cont. remiss.	1.57	FH	-	1–20%	<1%	71–100%
12	**Normoxic**	male	6	3	Thorax	Cont. remiss.	1.27	FH	-	1–20%	<1%	71–100%
13	**Normoxic**	female	11	3	Thorax	Resid. disease	1.31	FH	-	1–20%	21–50%	51–70%
14	**Normoxic**	male	146	3	Pelvis	Deceased	1.97	UH	+	1–20%	1–20%	51–70%
15	**Normoxic**	female	7	2B	Thorax	Cont. remiss.	na	FH	-	<1%	1–20%	71–100%
16	**Normoxic**	male	10	2B	Abdomen	Resid. disease	1.63	FH	-	1–20%	<1%	21–50%
17	**Normoxic**	female	10	2B	Pelvis	Cont. remiss.	1.46	FH	-	<1%	1–20%	71–100%
18	**Normoxic**	male	0.5	2A	Abdomen	Cont. remiss.	1.64	FH	-	1–20%	1–20%	71–100%
19	**Normoxic**	male	4	2A	Abdomen	Cont. remiss.	1.53	FH	-	1–20%	1–20%	71–100%
20	**Normoxic**	female	2	1	Abdomen-adrenal gland	Cont. remiss.	1.73	FH	-	<1%	1–20%	51–70%
21	**Normoxic**	male	3	1	Abdomen-adrenal gland	Cont. remiss.	1.34	FH	-	1–20%	1–20%	51–70%
22	**Normoxic**	male	3	1	Abdomen-adrenal gland	Cont. remiss.	1.75	FH	-	1–20%	<1%	71–100%
23	**Normoxic**	male	7	1	Abdomen-adrenal gland	Cont. remiss.	na	FH	-	21–50%	1–20%	71–100%
24	**Normoxic**	female	24	1	Thorax-abdomen	Resid. disease	1.66	FH	-	21–50%	21–50%	71–100%
25	**Normoxic**	female	53	1	Abdomen-retroperitoneal ganglia	Cont. remiss.	1.26	FH	-	51–70%	1–20%	71–100%

Clinicopathological characteristics of patients and tumor samples examined.

^1^ Outcome: Relapsed = relapsed NB after chemotherapy; Resid. Disease = residual NB after chemotherapy; Cont. remiss. = NB in continuous remission.

^2^ DNA ploidy is displayed as index of DNA content: 1 = diploidy; >1 = hyperdiploidy; <1 = hypodiploidy; na = not available.

^3^ Histology: UH = unfavorable FH = favorable.

^4^ N-myc amplification status: + = amplified; - = normal.

^5^ The protein expression level of HIF-1α, PDK1 and PHD3, obtained by immunohistochemical staining, is expressed as percentage of positive cells: <1% or 1–20% = low positivity; 21–50% = medium positivity; 51–70% or 71–100% = high positivity.

### NB cell lines and cultures

Human NB cell lines ACN, IMR-32 (ICLC-HTL96020 and ICLC-HTL96021 by ICLC-Interlab Cell Line Collection, IRCCS AOU San Martino-IST, Genova, Italy) and LAN-1 (ACC-655 by DSMZ-German Collection of Microorganisms and Cell Cultures, Leibniz Institute, Braunschweig, Germany), were cultured in either RPMI 1640 (Euroclone, Milano, Italy) or DMEM (Sigma-Aldrich, St Louis, MO, USA) supplemented with 10% FBS (Invitrogen, Carlsbad, CA, USA) and 2mM glutamine/penicillin-streptomycin. Cells were maintained at 37°C in a humidified incubator containing 20% O_2_, 5% CO_2_and 75% N_2_, referred to as normoxic condition. Alternatively, cells were maintained for 18, 72 or 96 hr before analysis in hypoxic conditions, obtained by cell incubation and handling in an anaerobic work-station incubator (Ruskinn Technology, Pencoed, UK) flushed with a mixture of 1% O_2_, 5% CO_2_ and 94% N_2_. Medium was allowed to equilibrate in the hypoxic incubator for at least 2 hr before use.

### Antibodies

Antibodies anti-human proteins used for Western blot (WB), immunocytochemistry (ICC) and immunohistochemistry (IHC-P) were: mouse monoclonal anti-VEGFA (sc-7269 by Santa Cruz Biotechnology, Dallas, TX, USA); rabbit polyclonal anti-PHD3 (ab30782 by Abcam, Cambridge, UK); mouse monoclonal anti-PDK1 for WB and ICC (ab110025 by Abcam); rabbit polyclonal anti-PDK1 for IHC-P (sc-28783 by Santa Cruz); rabbit polyclonal anti-PFKFB4 (PA5-15475 by Thermo Scientific, Waltham, MA, USA); mouse monoclonal anti-HIF-1α for WB (610959 by BD Biosciences, Franklin Lakes, NJ, USA); rabbit polyclonal anti-HIF-1α for ICC and IHC-P (NB100-479 by Novus Biologicals, Littleton, CO, USA); rabbit polyclonal anti-N-Myc (23960002 by Novus Biologicals).

### Western blot analysis

Human NB cells, grown in normoxic or hypoxic conditions, were lysed in a buffer containing 1,6 mM NaH_2_PO_4_, 8,6 mM Na_2_HPO_4_, 1% Triton X-100, 0,1% SDS, 0,1% NaN_3_, 0,1M NaCl, 0,5% NaDoc, 2 mM AEBSF, and 20 mg/ml each of aprotinin and leupeptin. Lysates (100 μg each) were subjected to 8% SDS-PAGE electrophoresis, transferred to PVDF membrane (Millipore, Billerica, MA, USA), and probed with anti-VEGFA (1:100), anti-PHD3 (1:1000), anti-PDK1 (1:500), anti-PFKFB4 (1:200), anti-HIF-1α (1:250), and anti-N-Myc (1:3000) antibodies. Proteins were visualized by West Dura extended chemiluminescent detection (Thermo Scientific) using HRP-conjugated secondary antibodies against mouse or rabbit (Pierce, Rockford, IL, USA). As loading control, blots were reprobed with mouse monoclonal anti-β-actin antibody (sc-47778 by Santa Cruz Biotechnology) (1:200). PBS and lysis-buffer were allowed to equilibrate in the hypoxic incubator for 1 hr before using them to wash and collect cells cultured in hypoxic conditions. (https://dx.doi.org/10.17504/protocols.io.jtvcnn6)

### Immunocytochemical analysis

Human NB cells were washed with PBS, detached from culturing flasks with trypsin, collected in PBS, counted, and cytospinned on Polysine™ slides (Bio Optica, Milano, Italy). PBS and trypsin were allowed to equilibrate in the hypoxic incubator for 1hr before using them to wash and detach cells cultured in 1% O_2_ conditions. Cells were then fixed in a methanol-acetone (1:1) solution for 15 minutes at -20°C and washed in PBS. Endogenous peroxidases were blocked with 3% H_2_O_2_ in distilled water for 15 minutes and non-specific binding was inhibited by incubating the samples in 20% goat serum (Sigma-Aldrich) in PBS-0,05% Tween (PBS-T), for 1 hr in a humidified chamber at 37°C. Cells were incubated for 2 hr at room temperature with each of the primary antibodies diluted in PBS-T as follows: anti-VEGFA (1:50), anti-PHD3 (1:250), anti-PDK1 (1:100), anti-PFKFB4 (1:50), anti-HIF-1α (1:150), and anti-N-Myc (1:200). Negative controls were made with PBS-T alone. Slides were washed with PBS and incubated first with biotinylated secondary goat antibody for 1 hr and then with HRP-streptavidin for 40 minutes (LSAB2 System-HRP kit, Dako, Glostrup, Denmark). The reaction was developed and visualized with DAB (Sigma-Aldrich) and counterstained with Harris’ hematoxylin (Bio Optica). Photographs were taken with the CX40 light microscope (Olympus, Tokyo, Japan) connected to the Olympus Altra-20 digital camera, and visualized with the AnalySIS^®^getIT imaging acquisition software. (https://dx.doi.org/10.17504/protocols.io.jtxcnpn)

### Histological and immunohistochemical analysis

NB specimens were fixed for 24–48 hr in 10% neutral buffered formalin (Bio Optica) and paraffin embedded. For histological analysis, 4 μm thick serial sections were stained with Harris’ hematoxylin and 1% eosin (Bio Optica). For immunohistochemistry experiments, serial sections were treated in a microwave oven three times for 5 min in citrate buffer (pH 6.0) at 750 W, then subjected to 3% H_2_O_2_ for 15 minutes and saturated with 20% goat serum in PBS-T, for 1 hr at 37°C. Samples were then incubated overnight at 4°C in a humidified chamber with the specific primary antibody diluted in PBS-T as follows: anti-VEGFA (1:50), anti-PHD3 (1:100), anti-PDK1 (1:100), anti-HIF-1α (1:150), and anti-N-Myc (1:200). Negative controls were made with PBS-T alone. After incubations with the biotinylated secondary antibody and the HRP-streptavidin (LSAB2 System-HRP kit, Dako), the reactions were developed and visualized as described above. The slides, which were blinded for specimen identification, were evaluated by a pathologist in a randomized order using a bright field microscope. Specimens were evaluated for the percentage of positive cells and for the staining intensity. The percentage of positive cells was confirmed by ImmunoRatio, a free, automated web-based image analysis application for scoring immune-stained slides [37]. (https://dx.doi.org/10.17504/protocols.io.jtxcnpn)

### Bioinformatics analysis

Formal analysis of the rules that characterize the association of the immunohistochemistry data (HIF-1α, PDK1, and PHD3) with the hypoxic status of the tumor defined by the microarray analysis was performed utilizing the APRIORI algorithm implemented by WEKA [38]. The expression level of each markers and the extent of hypoxia was dichotomized in high or low [39]. The rules performance was evaluated by the following parameters: coverage, confidence, lift, leverage, conviction. We selected seven rules with the highest lift or with the highest conviction, respectively. The resulting 14 highly significant rules are shown in Table 2.

**Table 2:** Association of the immunohistochemistry data with the hypoxic status of the tumor.

Rule	if HIF-1α is	andif PDK1 is	andif PHD3 is	then	Occurrence		Coverage	Confidence	Lift	Leverage	Conviction
					If	then					
R1	high			= = >Hypoxia = **high**	11	8	1.00	0.73	2.27	0.18	1.87
R2		high		= = >Hypoxia = **high**	9	7	0.88	0.78	2.43	0.16	2.04
R3			low	= = >Hypoxia = **high**	10	8	1.00	0.8	2.5	0.19	2.27
R4	high		low	= = >Hypoxia = **high**	8	8	1.00	1	3.13	0.22	5.44
R5	high	high		= = >Hypoxia = **high**	8	7	0.88	0.88	2.73	0.18	2.72
R6		high	low	= = >Hypoxia = **high**	7	7	0.88	1	3.13	0.19	4.76
R7	high	high	low	= = >Hypoxia = **high**	7	7	0.88	1	3.13	0.19	4.76
R8	low			= = >Hypoxia = **low**	14	14	0.82	1	1.47	0.18	4.48
R9		low		= = >Hypoxia = **low**	16	15	0.88	0.94	1.38	0.16	2.56
R10			high	= = >Hypoxia = **low**	15	15	0.88	1	1.47	0.19	4.8
R11	low	low		= = >Hypoxia = **low**	13	13	0.76	1	1.47	0.17	4.16
R12	low		high	= = >Hypoxia = **low**	12	12	0.71	1	1.47	0.15	3.84
R13		low	high	= = >Hypoxia = **low**	13	13	0.76	1	1.47	0.17	4.16
R14	low	low	high	= = >Hypoxia = **low**	11	11	0.65	1	1.47	0.14	3.52

Rules were generated by the APRIORI algorithm to associate the immunohistochemistry results for HIF-1α, PDK1 and PHD3 with the hypoxic status of the tumor.

## Results

### PDK1 and PHD3 protein expression is modulated in NB cell lines cultured under hypoxic conditions

We previously described a robust 62-probesets signature (NB-hypo) from microarray data, measuring the hypoxic status of NB cells and tumors. We found that NB-hypo stratifies NB patients on the bases of outcome [21]. On these premises, we have evaluated the possibility of utilizing hypoxia signature in immunohistochemistry on paraffin embedded NB tumor samples in order to define their hypoxic status.

**Fig 1:**
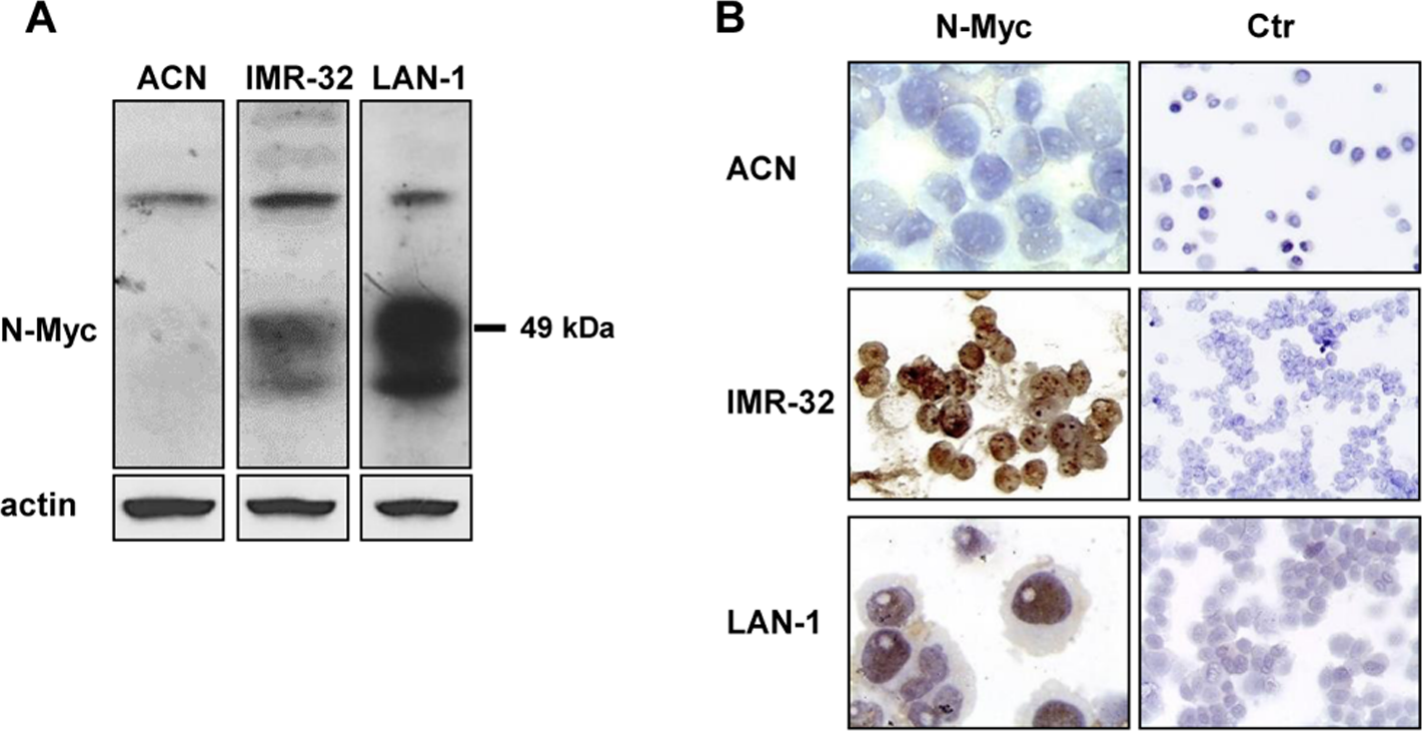
Expression levels and localization of N-Myc protein in NB cell lines. **(A)** Protein lysates from ACN, IMR-32, and LAN-1 cells were subjected to Western blot and probed with anti-N-Myc antibody. The blot was reprobed with anti-actin antibody as loading control. **(B)** ACN, IMR-32, and LAN-1 cells were cytospinned on Polysine™ slides and fixed. N-Myc protein nuclear localization was visualized by immunocytochemistry with anti-N-Myc antibody (60x magnification). Ctr = negative controls (20x magnification).

First, we selected 4 genes PDK1, PHD3, PFKFB4 and VEGFA proteins as representative of the NB-hypo based on the literature and strength of microarray signal and we validated the correspondence between protein expression and hypoxic state in NB cell lines. We chose three human NB cell lines: ACN, which displays no amplification of N-Myc, and IMR-32 and LAN-1, both characterized by N-Myc amplification (Fig 1A and 1B) [1]. Moreover, we analyzed the expression of HIF-1α, an accepted marker of low oxygen environment [29].

**Fig 2:**
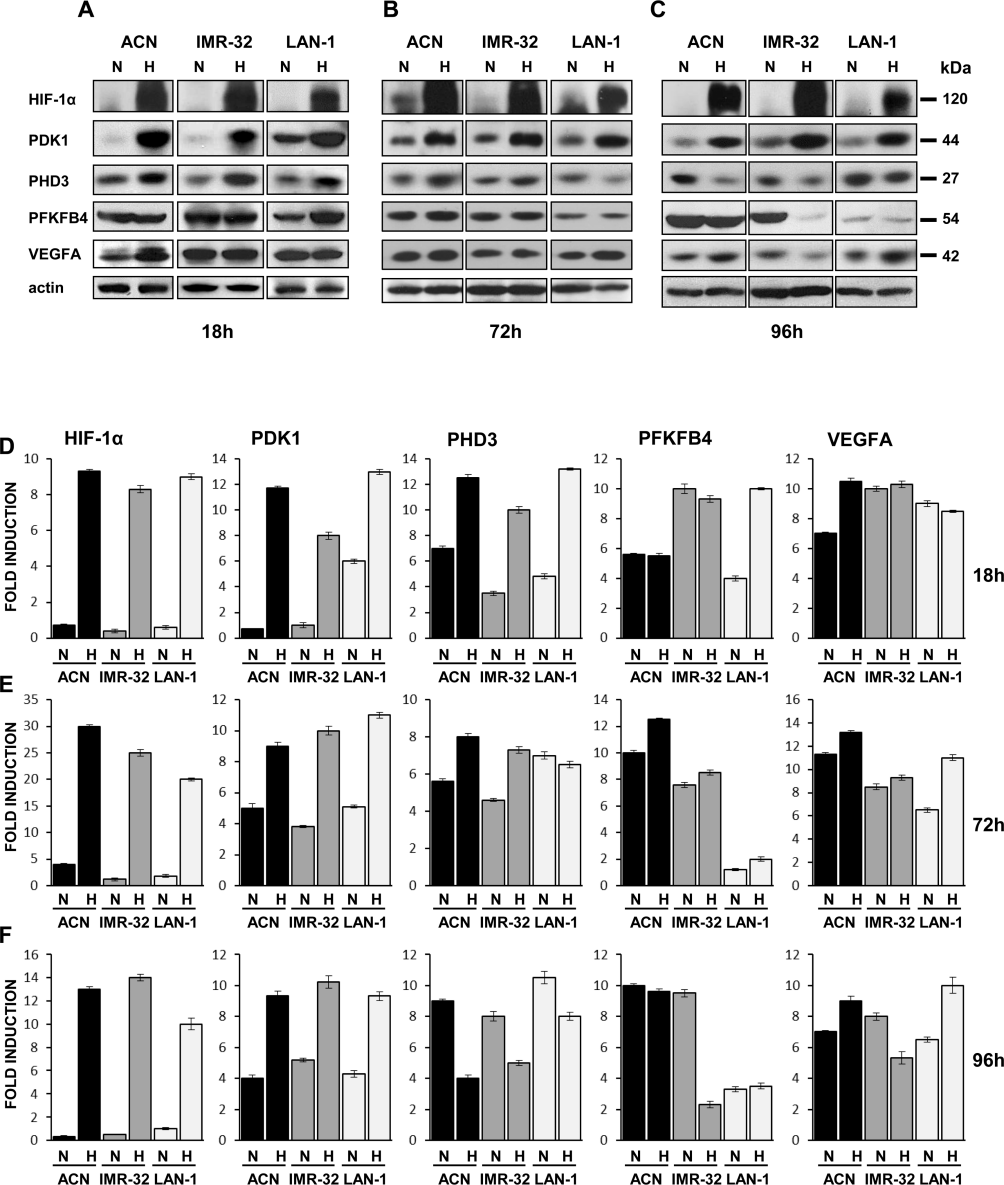
Hypoxia modulates expression of NB-hypo selected genes in NB cell lines. Protein lysates from ACN, IMR-32, and LAN-1 cells, cultured in normoxic (N) or in hypoxic (H) conditions for **(A)** 18, **(B)** 72, or **(C)** 96 hr, were subjected to Western blot and probed with anti-HIF-1α, anti-PDK1, anti-PHD3, anti-PFKFB4, and anti-VEGFA antibodies. The blot was reprobed with anti-actin antibody as loading control. Levels of HIF-1α, PDK1, PHD3, PFKFB4, and VEGFA in NB cells cultured in normoxic or in hypoxic conditions for **(D)** 18, **(E)** 72, or **(F)** 96 hr were quantified by densitometry and normalized to the content of the loading control protein. The optical density of the scanned film was measured with Quantity One v. 2–3 Image software (Versa Doc, Bio-Rad, Hercules, CA, USA). Results represent the mean values ± S.D. from three independent experiments.

NB cell lines were cultured under normoxic or hypoxic conditions for 18, 72, and 96 hr, harvested and lysed. Aliquots of cell lysates were then subjected to SDS-PAGE and immunoblotting, using specific antibodies against HIF-1α, PDK1, PHD3 PFKFB4 and VEGFA (Fig 2). As expected, culture under hypoxic condition stimulated the expression of HIF-1α. High expression of PDK1 was observed at all times of culture under hypoxic condition. Expression of PHD3 was transiently stimulated after 18 hr culture in low oxygen (Fig 2A and 2D), decreased after 72 hr (Fig 2B and 2E), and was further downregulated after 96 hr culture in hypoxia conditions (Fig 2C and 2F). The low expression of PHD3 protein observed after 96 hr culture in low oxygen is compatible with its function as an oncosuppressor and the knowledge that low levels of this enzyme correlate with poor outcome of different types of carcinomas [40,41]. PFKFB4 and VEGFA were variably modulated in each cell line. VEGFA expression was influenced by oxygen concentration only in ACN cells after 18 hr culture (Fig 2A and 2D) and in LAN-1 cells after 72 hr and 96 hr culture (Fig 2B, 2E and 2C and 2F), while PFKFB4 expression was upregulated by hypoxia in LAN-1 cells after 18 hr culture (Fig 2A and 2D) and downregulated in IMR-32 cells after 96 hr culture (Fig 2C and 2F). These results indicate that the expression of HIF-1α, PDK1, and PHD3 at the protein level in all the three cell lines is in agreement with the literature and the microarray results whereas PFKFB4 and VEGFA proteins expression was modulated by hypoxia at a different extent and times in the various cell lines and therefore poorly representative of the *in vivo* situation.

**Fig 3:**
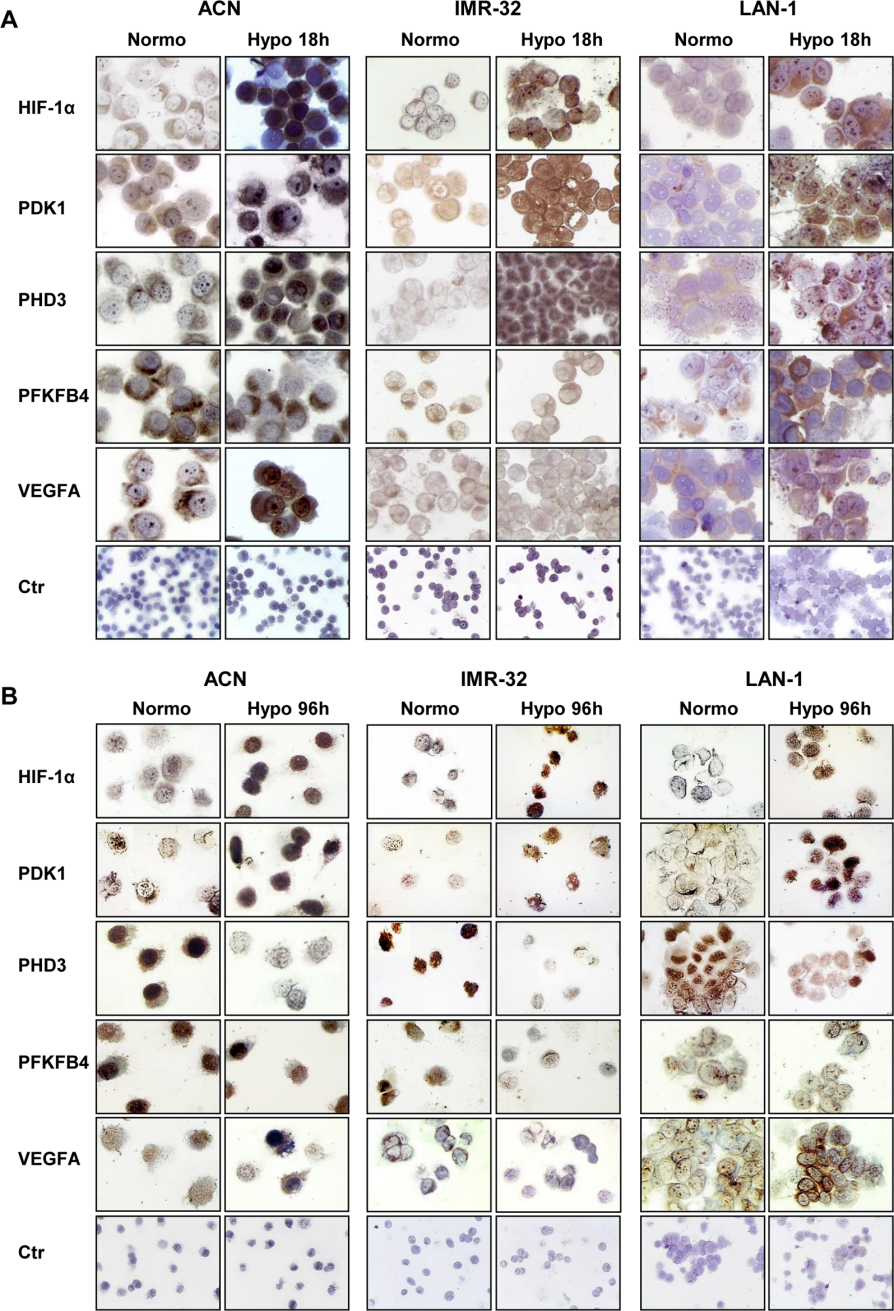
Immunocytochemical analysis of NB cells cultured in low oxygen concentration. ACN, IMR-32, and LAN-1 cells, cultured in normoxic (Normo) or in hypoxic conditions for **(A)** 18 (Hypo 18h) or **(B)** 96 (Hypo 96h) hr, were cytospinned on Polysine™ slides, fixed and treated with anti-HIF-1α, anti-PDK1, anti-PHD3, anti-PFKFB4, and anti-VEGFA antibodies (60x magnification). Ctr = negative controls (20x magnification).

We further analyzed the expression of HIF-1α, PDK1, PHD3, PFKFB4 and VEGFA in NB cell lines by immunocytochemistry. Cells were cultured for 18hr and 96 hr in hypoxic condition, detached, fixed on polylysine-coated slides, and treated with specific antibodies for immunocytochemistry (Fig 3). The results indicate that the expression of PFKFB4 and VEGFA was differently modulated by hypoxia in the various cell lines at 18 and 96 hr. On the other hand expression of HIF-1α and PDK1 was stimulated by exposure to hypoxic conditions, as observed in Western blot. HIF-1α, which is localized both in the nucleus and in the cytoplasm [29], and PDK1, which is distributed only in the cytoplasm [42], always display a more intense positivity when cells are cultured in low oxygen (Fig 3A and 3B). Moreover, hypoxia stimulates HIF-1α translocation from the cytosol to the nuclear compartment to exert its function [43]. PHD3, which localizes in the cytoplasm and nucleus following HIF-1α distribution [44], is upregulated upon 18 hr culture in hypoxia (Fig 3A), but is downregulated after 96 hr of hypoxic conditions (Fig 3B), again confirming Western blot observations. These results suggest that HIF-1α, PDK1, and PHD3 proteins may be used as markers of the hypoxic environment and the possibility of defining hypoxia signatures by immunohistochemistry on paraffin embedded tissues.

### Clinicopathological information of the NB tumor samples used for IHC staining of hypoxia markers

We consequently proceeded to investigate gene expression in human NB specimens. A total 25 NB tumor samples were collected. Tumors were selected from a normoxic and a hypoxic cluster on the basis of a microarray NB database, and divided in two groups, accordingly. The clinicopathological informations of the 25 pediatric patients are listed in Table 1.They were males and females with age at diagnosis between 15 days and 12 years. The onset anatomical site was the abdominal region for the large majority of the cases. In the hypoxic cluster, the INSS NB stage was mainly 4, with a metastatic tumor diffused to distant lymph nodes, bones, bone marrow, liver, skin and other organs and a general poor outcome. In the normoxic cluster, NB stages were 1, 2A, 2B and 3, localized tumors with or without involvement of regional lymph nodes and a general good outcome. Molecular features of the tumors were represented by N-myc amplification and ploidy evaluation. N-myc was amplified in 28% of the patients, the tumor ploidy was >2 in 8% of the patients and neither correlated with the hypoxic status. Seven patients showed unfavorable histology which was mostly associated with the presence of a hypoxic profile, consistent with previous findings demonstrating that hypoxia is an independent risk factor for neuroblastoma [18,21].

### The differential expression of HIF-1α, PDK1 and PHD3 in NB specimens represents a marker for tumor hypoxia environment

**Fig 4:**
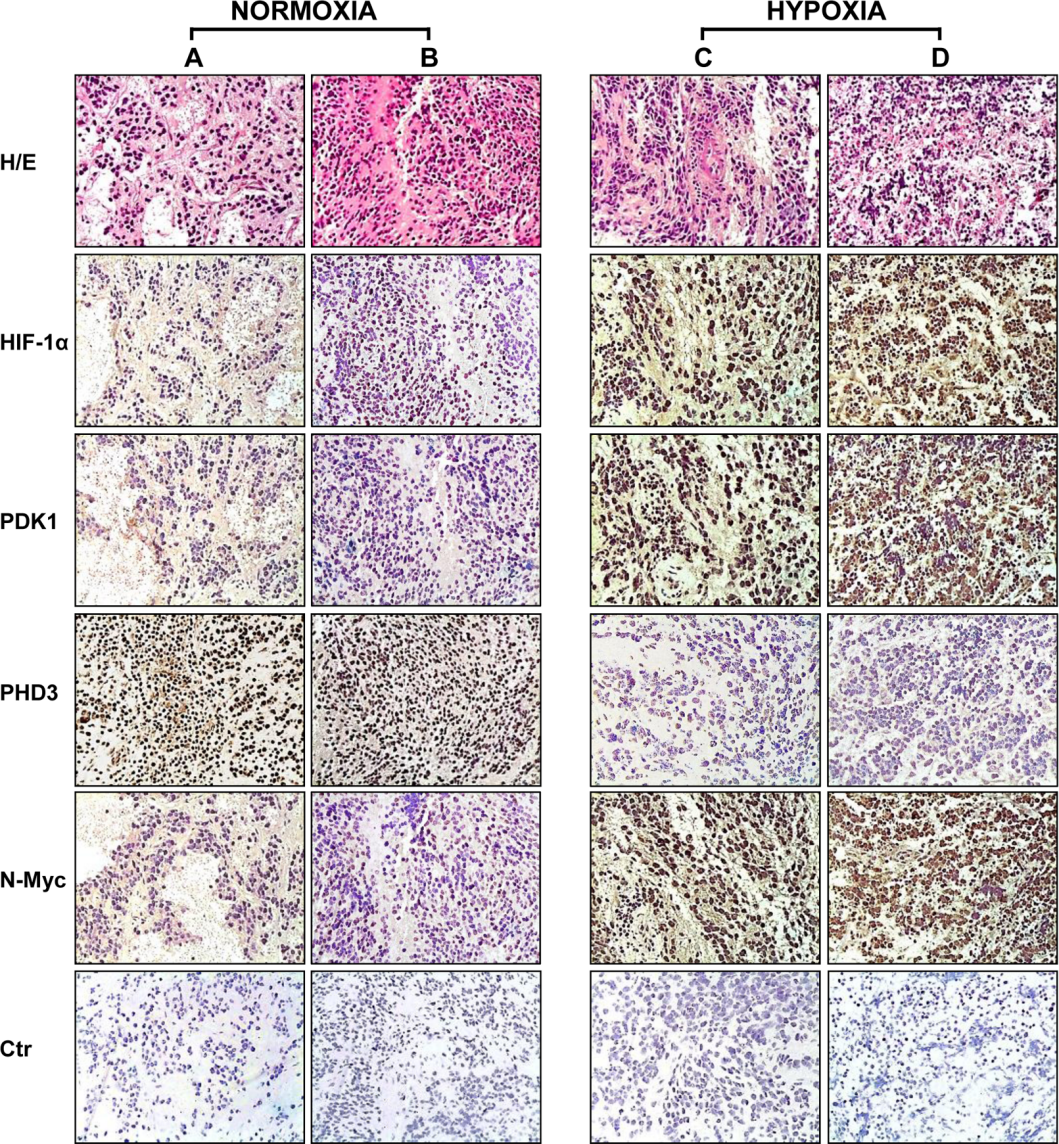
Immunohistochemical analysis of NB specimens for expression of NB-hypo selected genes. Tumor samples from the normoxic cluster **(A, B)** or from the hypoxic cluster **(C, D)** were fixed and paraffin embedded. **(A)** adrenal gland, stage 1 (Tumor number 22, Table 1); **(B)** mediastinal mass, stage 2B (Tumor number 15, Table 1); **(C)** abdominal mass, stage 4 (Tumor number 2, Table 1); **(D)** adrenal gland, stage 4 (Tumor number 3, Table 1). For histological analysis, sections were stained with hematoxylin and eosin (H/E). Serial sections were treated with anti-HIF-1α, anti-PDK1, anti-PHD3, and anti-N-Myc antibodies. Tumors with favorable prognosis **(A, B)** show negative staining for HIF-1α and PDK1, and positive staining for PHD3. Tumors with poor prognosis **(C, D)** display a positive staining for HIF-1α and PDK1, while they are almost negative for PHD3. N-Myc negativity in normoxic samples and N-Myc positivity in hypoxic ones is considered as a control for the accuracy of the immunohistochemical procedure. Ctr = negative controls. (20x magnification).

We performed immunohistochemical analysis on the 25 NB tumor specimens listed in Table 1 to evaluate HIF-1α, PDK1, and PHD3 protein expression. Serial sections from paraffin-embedded samples were stained with specific antibodies. Fig 4 shows the representative results obtained on two hypoxic and two normoxic samples. Normoxic samples (Fig 4A and 4B) derive from patients in continuous remission and they are, respectively, a stage 1 tumor with a 80% neoplasm, and a stage 2B with a 90% neoplasm. They both display a clear positivity for PHD3 but are negative for HIF-1α and PDK1. These results are compatible with the classification of these tumors as normoxic based on the cell lines’ results. Hypoxic samples C and D, derived from deceased patients, are stage 4 tumors with a 90% neoplasm. The hypoxic tumors are characterized by high expression of HIF-1α. As predicted, hypoxic tumors have high expression of PDK1 and low expression of PHD3 relative to normoxic tumors. N-Myc expression is negative in samples A and B and positive in samples C and D, in agreement with amplification data.

**Fig 5:**
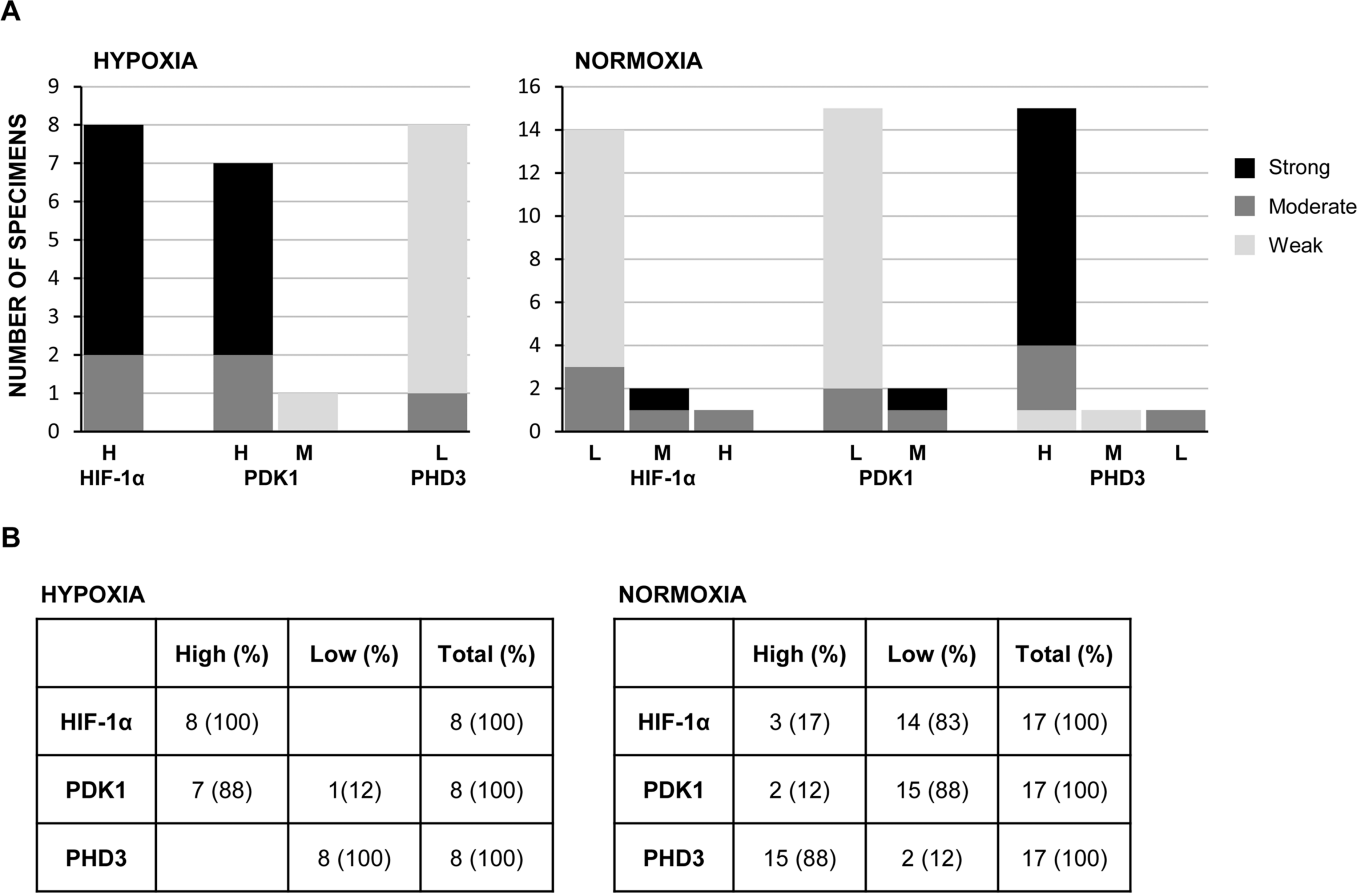
Evaluation of immunohistochemistry staining intensity of the tumor samples examined and listed in Table 1. **(A)** Each group of protein expression (L = low percentage of positive cells; M = medium percentage of positive cells; H = high percentage of positive cells) was further subdivided in strong, moderate or weak staining degree. **(B)** To calculate the total number of tumors with high or low positivity for each gene, the samples identified as M were considered H or L on the basis of staining intensity. The bioinformatics analysis were then performed as reported in Table 2.

The immunohistochemical pattern of expression of PHD3, PDK1, and HIF-1α in the NB specimens examined is summarized in Table 1 and in Fig 5. For each tumor specimen included in the study, we considered the percentage of positive cells (Table 1) and the staining intensity (Fig 5A). While most of the normoxic tumors show high expression of PHD3 and low expression of PDK1 and HIF-1α, and the majority of the hypoxic tumors display high expression of HIF-1α and PDK1 but low expression of PHD3, some variability was observed (Fig 5B and panels in S1 Fig and in S2 Fig).

The relationship among these variables was assessed utilizing association rules, a bioinformatic approach that outputs easily understandable plain language sentences. We utilized the APRIORI algorithm to derive relevant rules to associate histochemical biomarkers to the definition of hypoxic NB obtained by microarray analysis. Each rule performance is measured by several parameters (coverage, confidence, lift, leverage, conviction), each measuring the strength and validity of the rule. The algorithm generated several rules, and we selected 14 rules having the highest parameters of lift or conviction. The results are shown in Table 2. The rules are expressed in the form IF = = >THEN and ordered by those associated to high hypoxia and low hypoxia respectively. Rule R1 states that if HIF-1α is high then hypoxia is high. Similarly, high PDK1 in rule R2 or low PHD3 in rule R3 predict the high hypoxic state. These conclusions are intuitive based on previous considerations. However, we noticed that rule R1 is satisfied 11 times in the dataset but only 8 of them are correctly attributed to high hypoxia. The coverage is therefore maximum but the confidence is lower and the conviction low. Similar considerations apply to rules R2 and R3. We conclude that the individual markers are indicators of the hypoxic state of the tumor but the interpretation of the results is prone to error. In contrast, when we utilize two or more biomarkers to define immunohistochemically the hypoxic status, we find that the confidence is maximal, lift and conviction are very high, indicating a good performance of combination of biomarkers to define high hypoxia. The coverage decreases but different combinations of biomarkers, present in rules R1→R7, can cover the entire database. It is simpler to define the low hypoxia status, as individual or combinations of biomarkers appear to have a similar high performance.

These data provide evidence that PDK1 and PHD3, together with HIF-1α, can be utilized as markers for the classification of NB tumors and demonstrate the possibility of assessing the hypoxic status by immunohistochemistry on paraffin embedded tissues.

## Discussion

Molecular tumor markers hold considerable promise for accurately predicting the progression of the disease in patients. However, in the majority of cases, single marker analysis has been found to have low robustness and it is of little use in clinical practice.

The present study investigated, by immunohistochemical analysis, the protein expression of HIF-1α, PDK1, PHD3, PFKFB4, and VEGFA, five genes extensively modulated in hypoxic conditions [21], the typical state of tumor microenvironment strictly connected with cancer cells growth and propagation [22–24,45]. Our results reveal that the differential expression of PDK1, PHD3, and HIF-1α in NB specimens can be used as hypoxia biomarkers in NB patients. PFKFB4 and VEGFA, on the other hand, were not evaluated in tumor specimens because of the variability and unpredictability of their expression in response to hypoxia in the three NB cell lines examined.

We explored the protein expression of these genes in NB cell lines and found that PDK1 and PHD3, together with HIF-1α, the distinctive biomarker for the low oxygen condition [29], were modulated in cells cultured in hypoxia. In particular, PDK1, the enzyme that enables cells to switch from mitochondrial respiration to anaerobic glycolysis [31], presented a constant very high expression in hypoxic conditions. PHD3, the enzyme that hydroxylates HIF-1α, causing its degradation [33], was higher than its normoxic level after 18 hours of oxygen deprivation, decreased to levels comparable to its expression in normoxic conditions after 72 hours, but was sensibly downregulated after 96 hours in low oxygen. We do not know the reason for the high expression of PHD3 in NB cells after 18 hr of culture in hypoxic condition followed by a switch to low expression, but a certain length of exposure to hypoxia may probably be necessary for the response of this gene to low oxygen, a condition that may mimic cancer adaptation to the hypoxic environment.

Recent reports indicate that hypoxia-inducible genes are associated with a poor prognosis in NB [15,18,46]. The NB specimens we used in this study were previously classified as hypoxic or normoxic and clustered on the basis of our NB-hypo genes signature [21], thus classifying them as high or low risk tumors, respectively. All the samples examined had at least 80%-90% of NB tumor cells. Most of the tumors classified as normoxic showed low expression of HIF-1α and PDK1 and high protein levels of PHD3, while the majority of the tumors classified as hypoxic displayed high positivity for HIF-1α and PDK1, and very low expression of PHD3.

Hypoxia induces broad adaptive changes in cell metabolism that are coordinated by HIF-1α at the transcriptional level [47]. Activation of HIF-1α occurs in several tumors, and primary tumors with low oxygenation are associated with an increased risk of metastasis and patient mortality [48].

HIF-1α-induced PDK1 plays a pivotal role in regulating mitochondrial activity. PDK1 acts as a gate-keeping mitochondrial enzyme that downregulates PDH activity, decreases the oxidation of pyruvate in mitochondria, and increases the conversion of pyruvate to lactate in the cytosol. Increased tumorigenicity correlates with higher PDK1 activity, lower PDH activity, and a dependence on glycolytic pathways. An elevated expression of PDK1 enzyme has been reported in several aggressive cancer types, such as gastric cancer [49], lung cancer [50], and myeloma [51]. Involvement of PDK1 in NB growth has been suggested by the fact that the activity of a glycolytic inhibitor that leads to lower levels of expression of PDK1 gene is correlated with suppression of growth of NB xenografts in mice [52]. Therefore, the high expression of PDK1 in tumor specimens classified as hypoxic is compatible with the aggressiveness displayed by these tumors.

In well-oxygenated environments, HIF-1α becomes hydroxylated by members of the PHD family (PHD1, PHD2, PHD3), and is targeted for degradation by the proteasome, while, under low oxygen availability, PHDs are inactive and HIF-1α translocates to the nucleus where it can regulate the transcription of target genes. Although the role of PHDs in cancer has been less studied, altered levels of PHD1, PHD2 and PHD3 have been correlated with the development of different types of carcinomas [40,41]. Thus, PHD3 appears to have oncosuppressive properties in many different tumors, with low levels of the enzyme correlating with poor outcome. This is the case of pancreatic, colorectal, and breast cancers [53], plasma cell neoplasia [54], pheochromocytoma [55] and glioblastoma [56], where PHD3 regulates neuronal apoptosis. PDH3, together with its downstream effectors, defines a pathway that is responsible for the elimination of excess neuroblasts during normal embryological development when growth factors, such as NGF, become limiting. Therefore, downregulation of PHD3 or its downstream effectors may lead to the pathogenesis of certain neural crest-derived tumors, such as NB [57]. Our study indicates that this gene is downregulated both in NB cells cultured in low oxygen and in hypoxic NB tumors, consistent with inhibition of its oncosuppressive activity and tumor growth and aggressiveness.

We demonstrated that tissue hypoxia is an independent risk factor for NB patients. A correlation between low oxygen tension and poor prognosis was reported for other tumor types. However, determination of oxygen levels in tissues is not an easy task and it relies on indirect markers activated by hypoxia. We demonstrated that a gene expression signature composed by 32 transcripts allowed the determination of the hypoxic status of the tumor and it was a strong, independent outcome predictor for NB patients [18,21]. The microarray approach to determine the hypoxic status of a tumor may not always be feasible because of the lack of fresh or frozen tumor specimens, the lack of the molecular technology or the bioinformatics expertise, and the high cost of this analysis. On the other hand, paraffin-embedded tumor tissues are always available and immunohistochemistry is a simple, less costly, and to everyone's reach technique. A new indicator of tissue hypoxia derived from specimens readily available in every tumor would improve dramatically our ability to define the hypoxic status of tumor tissues in a broad cohort of patients. Examination of the paraffin embedded tissue from primary tumors by the pathologist is the first mandatory step for tumor characterization. Therefore, the use of paraffin embedded tissue for tumor characterization allows retrospective studies of large tumor datasets. The definition of tumor hypoxia by immunohistochemistry on tumor slides would allow the assignment of a label for the hypoxic status in many NB tumors where the frozen sample is not available. We found that a three protein signature, detectable by immunohistochemistry and analyzed by the appropriate algorithm, was able to assess the oxygen level of NB tumors, splitting them in normoxic and hypoxic tissues. Therefore, we will not have to rely on the availability of large biopsies for tissue hypoxia determination but we can obtain this information from a few tumor slices. The three protein-defined hypoxic status may become one additional risk factor easily determined to improve the prognosis of NB tumors. Moreover, paraffin embedded tissues are saved for several years by the pathologists and retrospective analysis can be performed to build a large dataset. Neuroblastoma is a rare pediatric tumor and the progress in understanding the evolution of the disease relies on extensive characterization of each tumor specimen. A large dataset will allow addressing question on the relationship among hypoxic status and known and established molecular risk factors such as N-myc amplification, ploidy evaluation or clinical indicators such as age at diagnosis or INSS stage. On these bases, we propose to use the three protein signature as a molecular correlate to measure NB tumor tissue hypoxia. This information will be instrumental to improve, for example, the stratification of high risk patients representing about 50% of the tumors with poor prognosis. We are currently working in expanding our dataset to include the hypoxic status of the tumor to be added to the other known markers. It may be that our three protein signature will detect the hypoxic status of other tumor types and this possibility is under evaluation. Finally, the APRIORI algorithm provides a very straightforward way to associate the results of the modulation of the three proteins with the tumor hypoxia, thereby allowing an easy reading of the combination of the results obtained with each determination.

In conclusion, our results indicate that the analysis of the expression of PDK1, PHD3, and HIF-1α by immunohistochemistry can be used to define the hypoxic status of NB tumors, which is a novel and important prognostic indicator to stratify high risk patients.

## Supporting information

S1 Fig Immunohistochemical analysis of the NB specimens examined from the hypoxic cluster. Histology (H/E) and immunohistochemical results for HIF-1α, PDK1, PHD3 and N-myc are shown for the other 6 tumor samples of the hypoxic cluster examined. The numbers on the left correspond to the tumor samples listed in Table 1. Ctr = negative controls. (20x magnification).
(TIF)

S2 Fig Immunohistochemical analysis of the NB specimens examined from the normoxic cluster. Histology (H/E) and immunohistochemical results for HIF-1α, PDK1, PHD3 and N-myc are shown for the other 15 tumor samples of the normoxic cluster examined. The numbers on the left correspond to the tumor samples listed in Table 1. Ctr = negative controls. (20x magnification).
(TIF)

S1 File Primary data for histograms. Primary data (mean values and standard deviations) obtained for the optical densities measured on the Western blot bands used to build the related histograms shown in Fig 2D, 2E and 2F.
(XLSX)

S2 File Original Western blots. Original unadjusted Western blot films, subdivided in three folders corresponding to each of the hypoxic conditions analyzed, i.e. growth of cells in hypoxia for 18, 72, and 96 hrs.
(ZIP)
