# Supplementary figures and images for: Immunohistochemical analysis of PDK1, PHD3 and HIF-1α expression defines the hypoxic status of neuroblastoma tumors
Source: PLoS One. 2017 Nov 8;12(11):e0187206. doi: 10.1371/journal.pone.0187206 (PMC5678880; doi:10.1371/journal.pone.0187206)

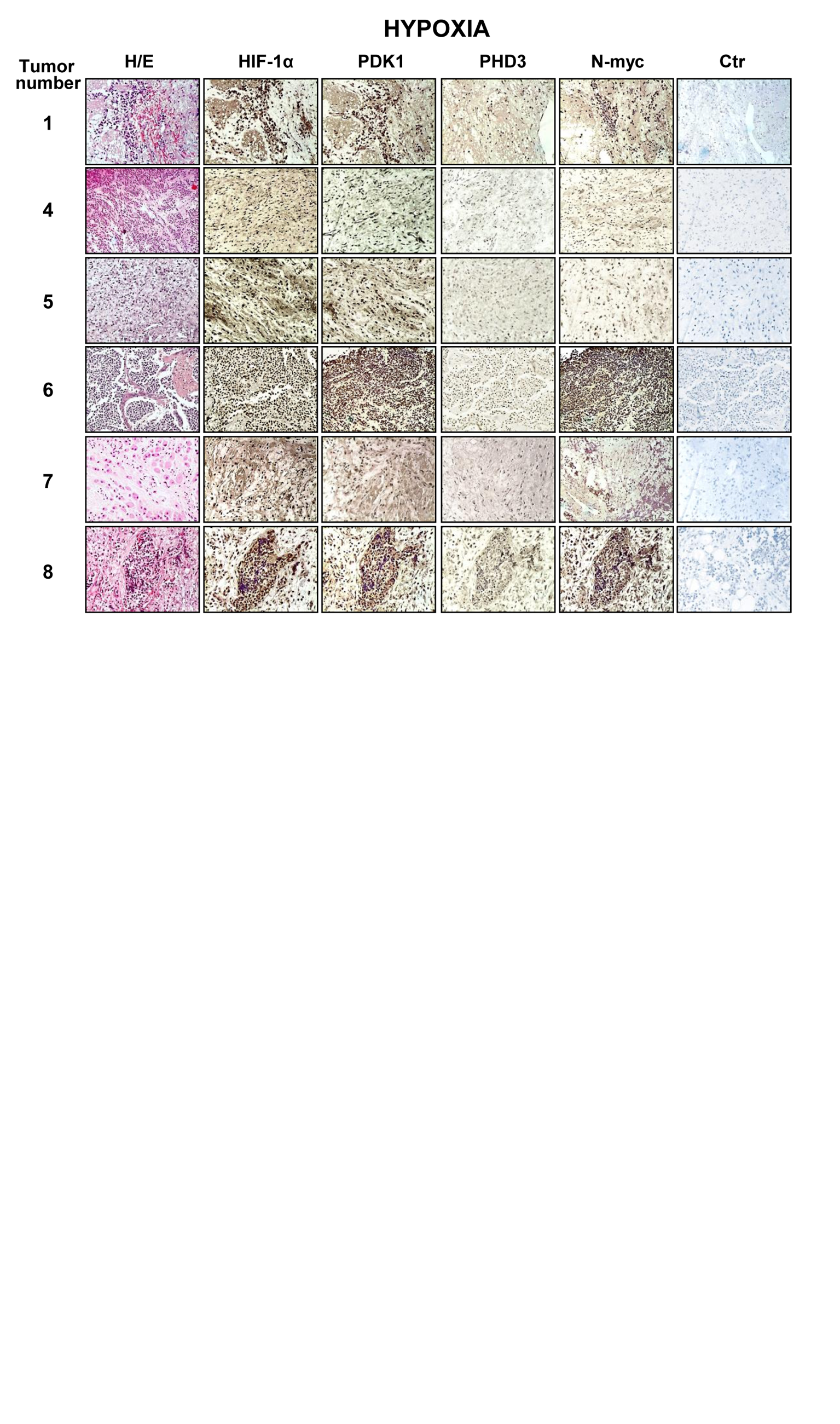

Supplement: S1 Fig — Histology (H/E) and immunohistochemical results for HIF-1α, PDK1, PHD3 and N-myc are shown for the other 6 tumor samples of the hypoxic cluster examined. The numbers on the left correspond to the tumor samples listed in Table 1. Ctr = negative controls. (20x magnification). (TIF) [file pone.0187206.s001.tif]

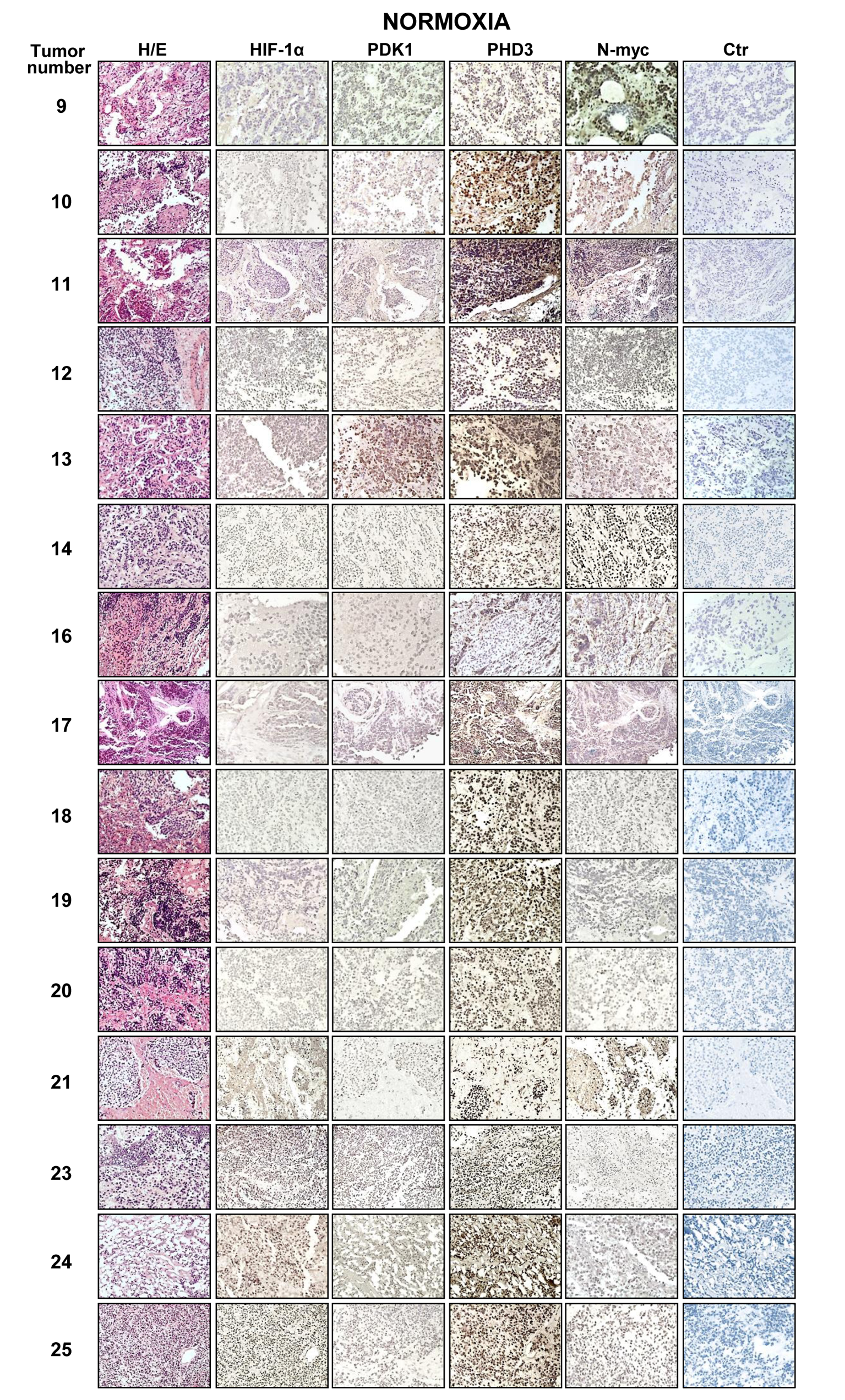

Supplement: S2 Fig — Histology (H/E) and immunohistochemical results for HIF-1α, PDK1, PHD3 and N-myc are shown for the other 15 tumor samples of the normoxic cluster examined. The numbers on the left correspond to the tumor samples listed in Table 1. Ctr = negative controls. (20x magnification). (TIF) [file pone.0187206.s002.tif]

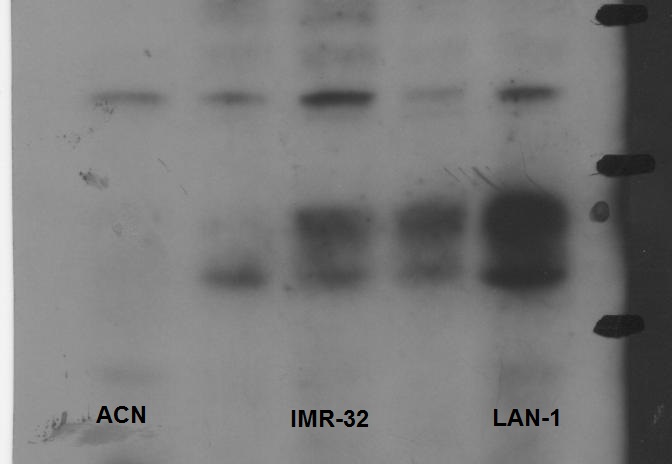

Supplement: S2 File — Original unadjusted Western blot films, subdivided in three folders corresponding to each of the hypoxic conditions analyzed, i.e. growth of cells in hypoxia for 18, 72, and 96 hrs. (ZIP) [file pone.0187206.s004.zip › 18h/01)n-myc.jpg]

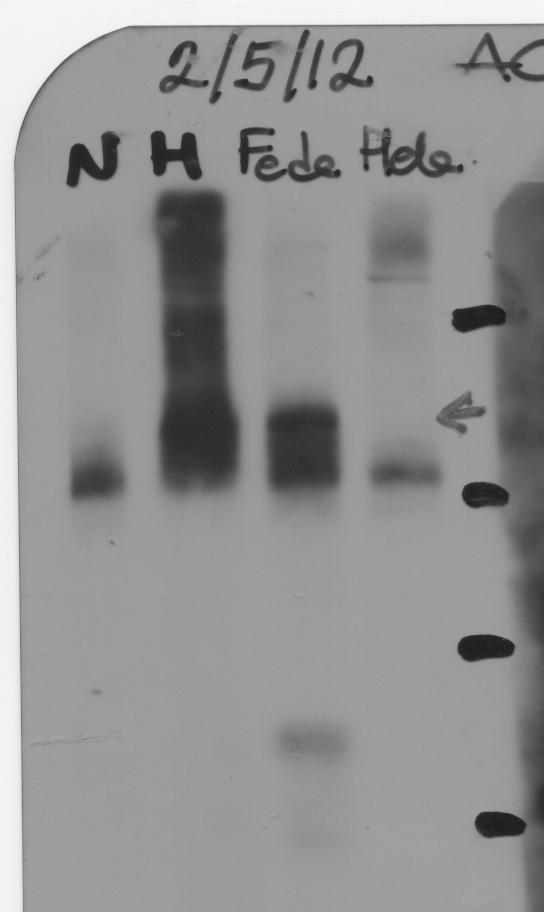

Supplement: S2 File — Original unadjusted Western blot films, subdivided in three folders corresponding to each of the hypoxic conditions analyzed, i.e. growth of cells in hypoxia for 18, 72, and 96 hrs. (ZIP) [file pone.0187206.s004.zip › 18h/02)hif ACN 18.jpg]

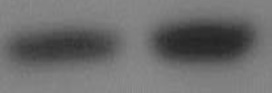

Supplement: S2 File — Original unadjusted Western blot films, subdivided in three folders corresponding to each of the hypoxic conditions analyzed, i.e. growth of cells in hypoxia for 18, 72, and 96 hrs. (ZIP) [file pone.0187206.s004.zip › 18h/03)phd3 ACN 18.jpg]

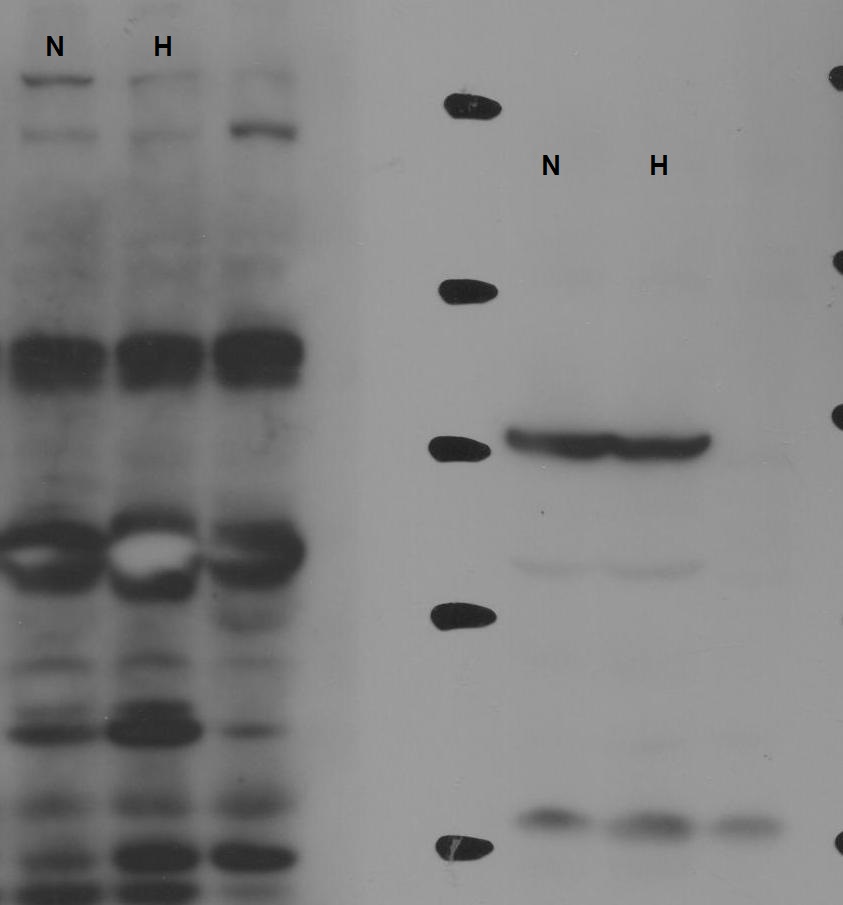

Supplement: S2 File — Original unadjusted Western blot films, subdivided in three folders corresponding to each of the hypoxic conditions analyzed, i.e. growth of cells in hypoxia for 18, 72, and 96 hrs. (ZIP) [file pone.0187206.s004.zip › 18h/04)vegf and pfk ACN 18.jpg]

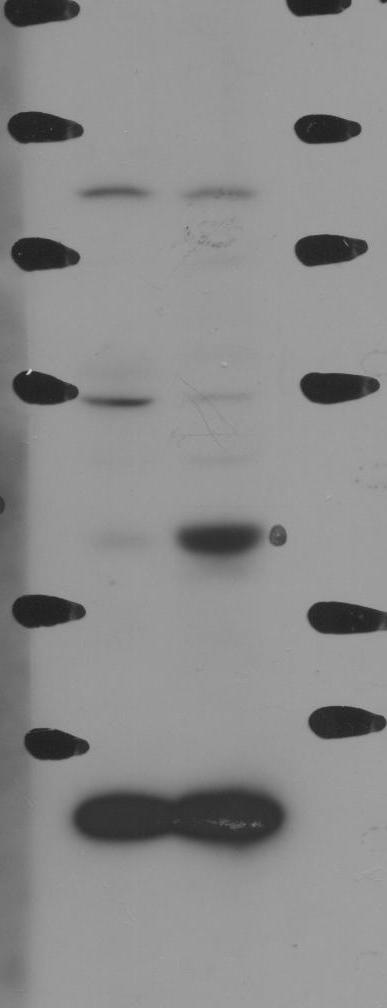

Supplement: S2 File — Original unadjusted Western blot films, subdivided in three folders corresponding to each of the hypoxic conditions analyzed, i.e. growth of cells in hypoxia for 18, 72, and 96 hrs. (ZIP) [file pone.0187206.s004.zip › 18h/05)pdk ACN 18.jpg]

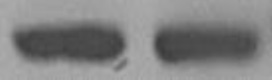

Supplement: S2 File — Original unadjusted Western blot films, subdivided in three folders corresponding to each of the hypoxic conditions analyzed, i.e. growth of cells in hypoxia for 18, 72, and 96 hrs. (ZIP) [file pone.0187206.s004.zip › 18h/06)actin ACN 18.jpg]

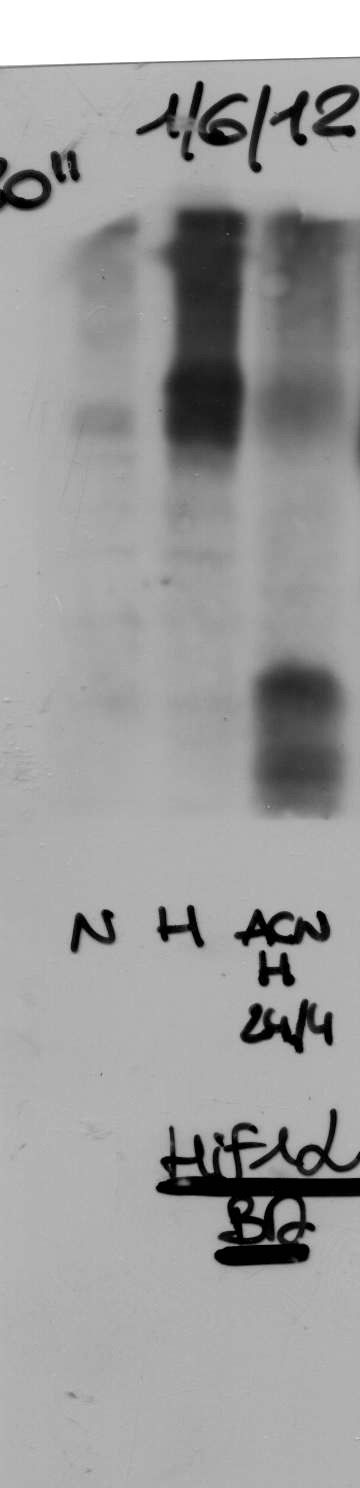

Supplement: S2 File — Original unadjusted Western blot films, subdivided in three folders corresponding to each of the hypoxic conditions analyzed, i.e. growth of cells in hypoxia for 18, 72, and 96 hrs. (ZIP) [file pone.0187206.s004.zip › 18h/07)hif IMR 18.jpg]

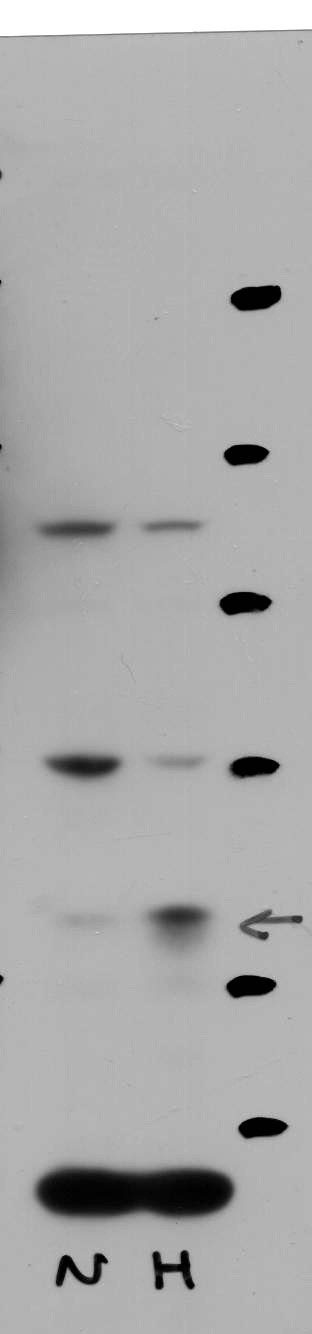

Supplement: S2 File — Original unadjusted Western blot films, subdivided in three folders corresponding to each of the hypoxic conditions analyzed, i.e. growth of cells in hypoxia for 18, 72, and 96 hrs. (ZIP) [file pone.0187206.s004.zip › 18h/08) pdk IMR 18.jpg]

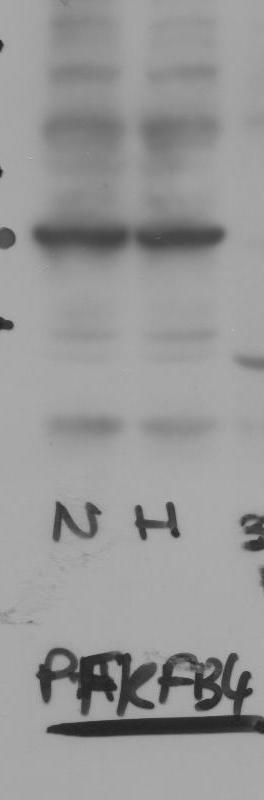

Supplement: S2 File — Original unadjusted Western blot films, subdivided in three folders corresponding to each of the hypoxic conditions analyzed, i.e. growth of cells in hypoxia for 18, 72, and 96 hrs. (ZIP) [file pone.0187206.s004.zip › 18h/09)pfk IMR 18.jpg]

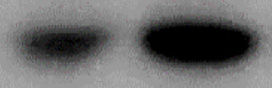

Supplement: S2 File — Original unadjusted Western blot films, subdivided in three folders corresponding to each of the hypoxic conditions analyzed, i.e. growth of cells in hypoxia for 18, 72, and 96 hrs. (ZIP) [file pone.0187206.s004.zip › 18h/10)phd3 IMR 18.jpg]

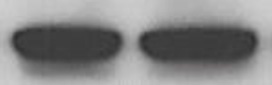

Supplement: S2 File — Original unadjusted Western blot films, subdivided in three folders corresponding to each of the hypoxic conditions analyzed, i.e. growth of cells in hypoxia for 18, 72, and 96 hrs. (ZIP) [file pone.0187206.s004.zip › 18h/10a)vegf IMR 18.jpg]

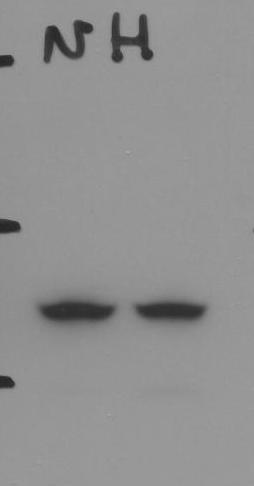

Supplement: S2 File — Original unadjusted Western blot films, subdivided in three folders corresponding to each of the hypoxic conditions analyzed, i.e. growth of cells in hypoxia for 18, 72, and 96 hrs. (ZIP) [file pone.0187206.s004.zip › 18h/10b)actin IMR 18.jpg]

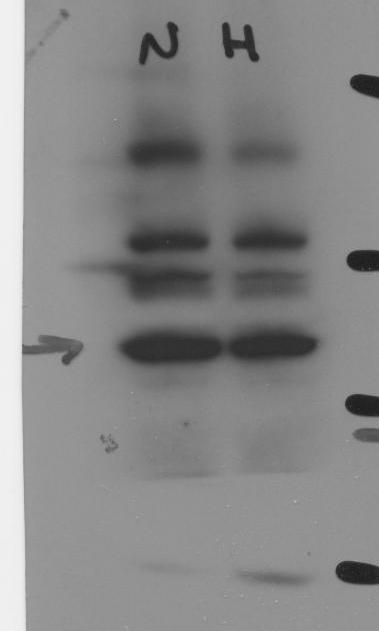

Supplement: S2 File — Original unadjusted Western blot films, subdivided in three folders corresponding to each of the hypoxic conditions analyzed, i.e. growth of cells in hypoxia for 18, 72, and 96 hrs. (ZIP) [file pone.0187206.s004.zip › 18h/11)vegf LAN 18.jpg]

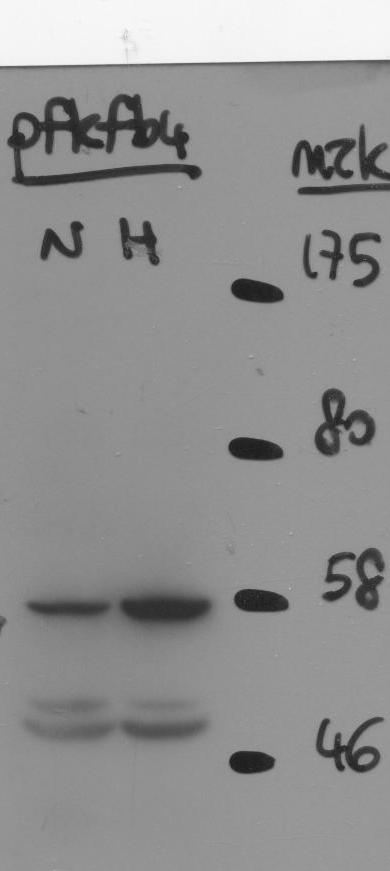

Supplement: S2 File — Original unadjusted Western blot films, subdivided in three folders corresponding to each of the hypoxic conditions analyzed, i.e. growth of cells in hypoxia for 18, 72, and 96 hrs. (ZIP) [file pone.0187206.s004.zip › 18h/12)pfk LAN 18.jpg]

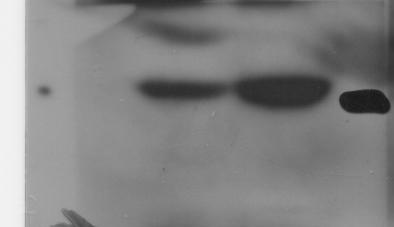

Supplement: S2 File — Original unadjusted Western blot films, subdivided in three folders corresponding to each of the hypoxic conditions analyzed, i.e. growth of cells in hypoxia for 18, 72, and 96 hrs. (ZIP) [file pone.0187206.s004.zip › 18h/13)pdk LAN 18.jpg]

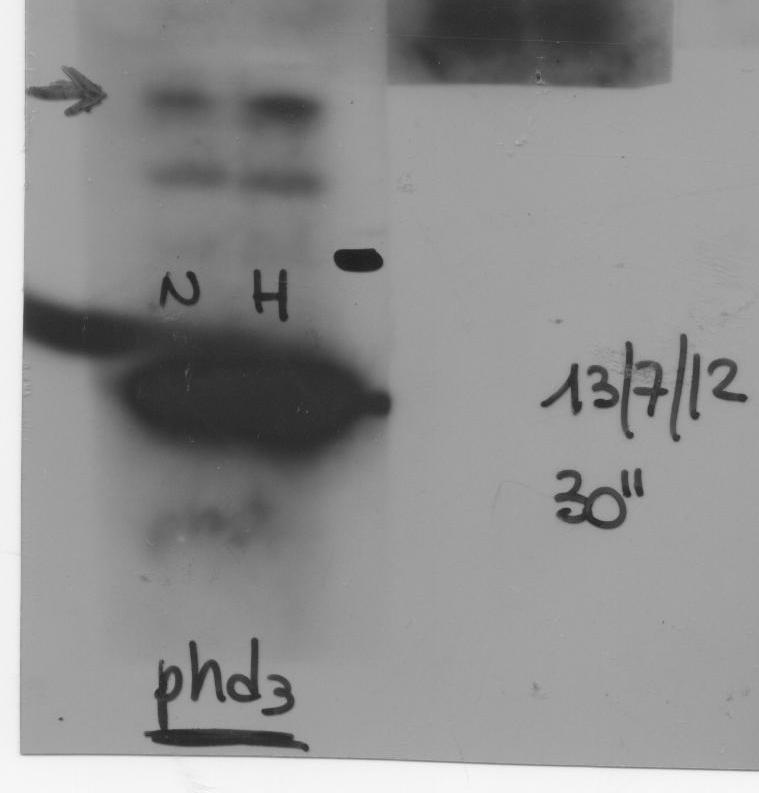

Supplement: S2 File — Original unadjusted Western blot films, subdivided in three folders corresponding to each of the hypoxic conditions analyzed, i.e. growth of cells in hypoxia for 18, 72, and 96 hrs. (ZIP) [file pone.0187206.s004.zip › 18h/13)phd3 LAN 18.jpg]

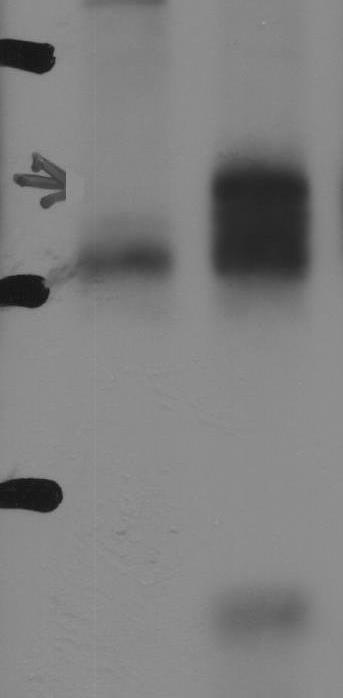

Supplement: S2 File — Original unadjusted Western blot films, subdivided in three folders corresponding to each of the hypoxic conditions analyzed, i.e. growth of cells in hypoxia for 18, 72, and 96 hrs. (ZIP) [file pone.0187206.s004.zip › 18h/14)hif LAN 18.jpg]

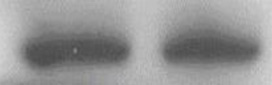

Supplement: S2 File — Original unadjusted Western blot films, subdivided in three folders corresponding to each of the hypoxic conditions analyzed, i.e. growth of cells in hypoxia for 18, 72, and 96 hrs. (ZIP) [file pone.0187206.s004.zip › 18h/15)actin LAN 18.jpg]

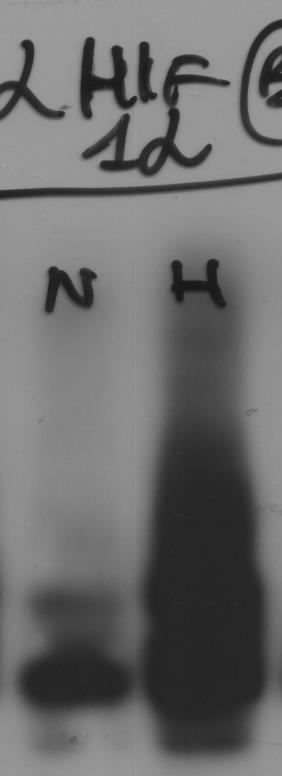

Supplement: S2 File — Original unadjusted Western blot films, subdivided in three folders corresponding to each of the hypoxic conditions analyzed, i.e. growth of cells in hypoxia for 18, 72, and 96 hrs. (ZIP) [file pone.0187206.s004.zip › 72h/01)hif ACN 72h.jpeg]

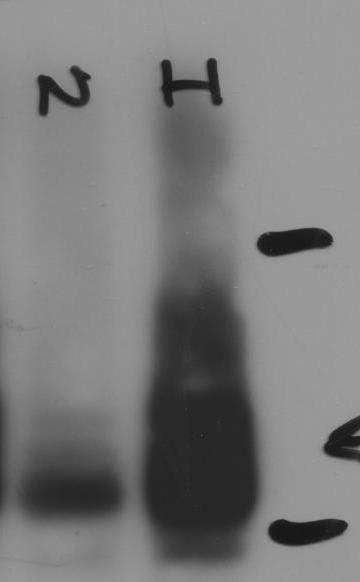

Supplement: S2 File — Original unadjusted Western blot films, subdivided in three folders corresponding to each of the hypoxic conditions analyzed, i.e. growth of cells in hypoxia for 18, 72, and 96 hrs. (ZIP) [file pone.0187206.s004.zip › 72h/02)hif IMR 72.jpeg]

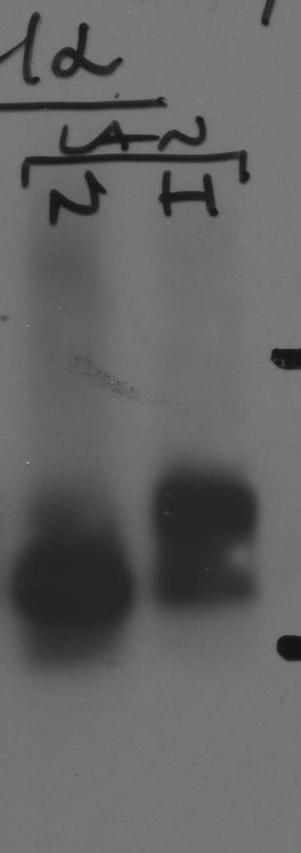

Supplement: S2 File — Original unadjusted Western blot films, subdivided in three folders corresponding to each of the hypoxic conditions analyzed, i.e. growth of cells in hypoxia for 18, 72, and 96 hrs. (ZIP) [file pone.0187206.s004.zip › 72h/03)hif LAN 72h.jpeg]

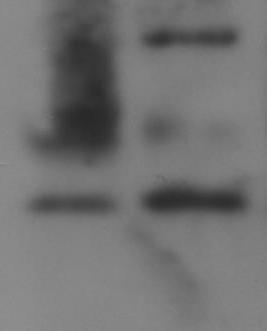

Supplement: S2 File — Original unadjusted Western blot films, subdivided in three folders corresponding to each of the hypoxic conditions analyzed, i.e. growth of cells in hypoxia for 18, 72, and 96 hrs. (ZIP) [file pone.0187206.s004.zip › 72h/07)pdk ACN 72.jpeg]

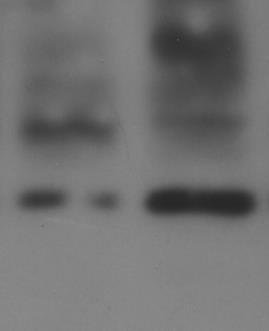

Supplement: S2 File — Original unadjusted Western blot films, subdivided in three folders corresponding to each of the hypoxic conditions analyzed, i.e. growth of cells in hypoxia for 18, 72, and 96 hrs. (ZIP) [file pone.0187206.s004.zip › 72h/08)pdk IMR 72.jpeg]

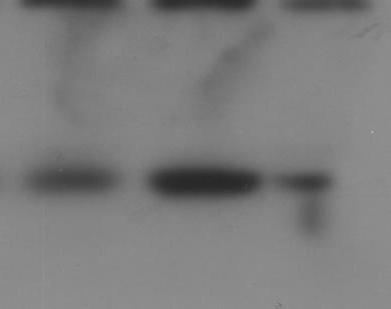

Supplement: S2 File — Original unadjusted Western blot films, subdivided in three folders corresponding to each of the hypoxic conditions analyzed, i.e. growth of cells in hypoxia for 18, 72, and 96 hrs. (ZIP) [file pone.0187206.s004.zip › 72h/09)pdk LAN 72.jpeg]

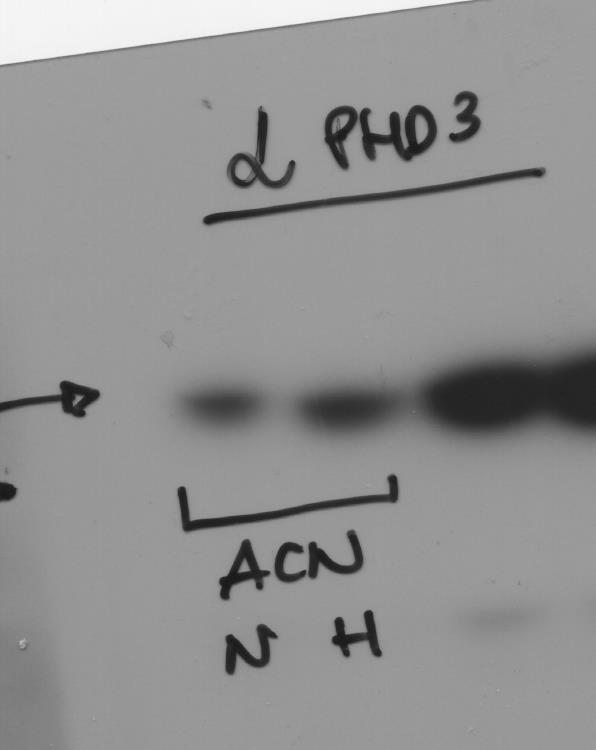

Supplement: S2 File — Original unadjusted Western blot films, subdivided in three folders corresponding to each of the hypoxic conditions analyzed, i.e. growth of cells in hypoxia for 18, 72, and 96 hrs. (ZIP) [file pone.0187206.s004.zip › 72h/11)phd3 ACN 72.jpeg]

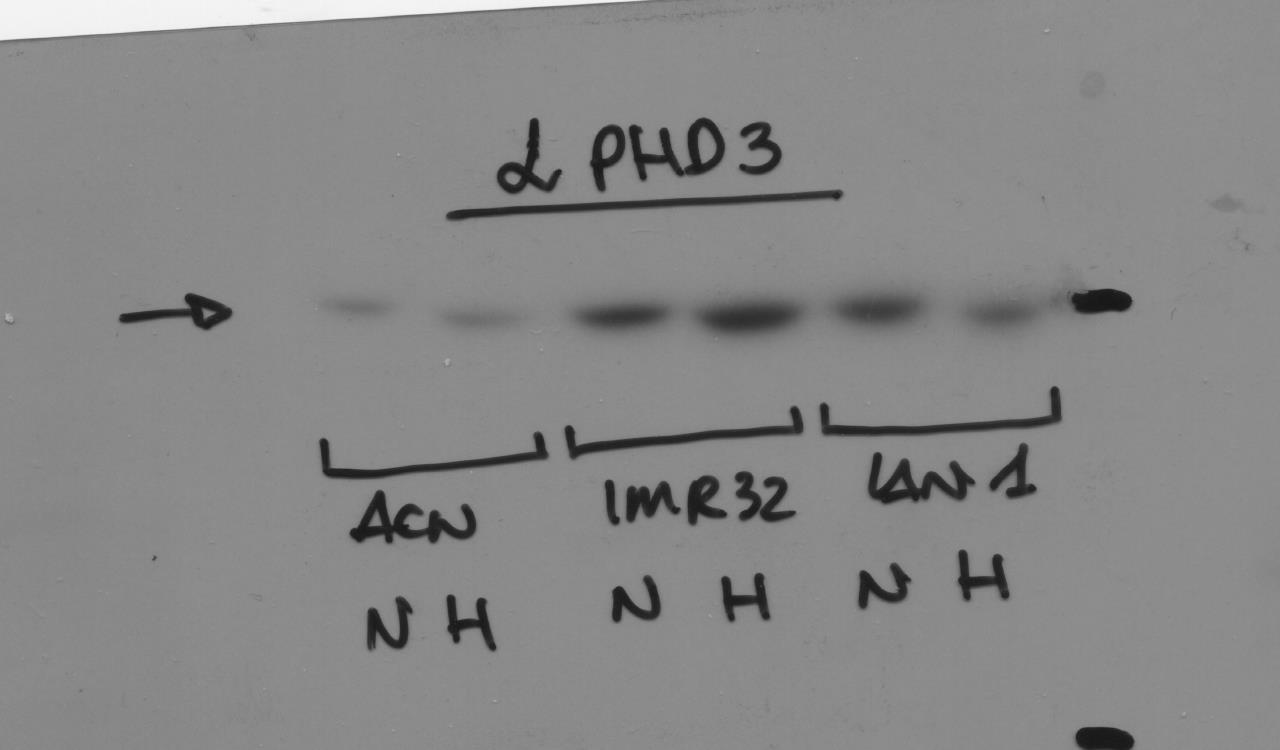

Supplement: S2 File — Original unadjusted Western blot films, subdivided in three folders corresponding to each of the hypoxic conditions analyzed, i.e. growth of cells in hypoxia for 18, 72, and 96 hrs. (ZIP) [file pone.0187206.s004.zip › 72h/12)phd3 IMR-LAN 72.jpeg]

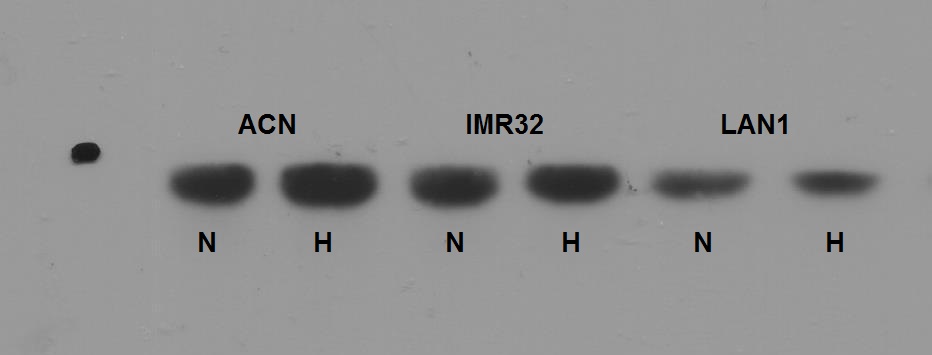

Supplement: S2 File — Original unadjusted Western blot films, subdivided in three folders corresponding to each of the hypoxic conditions analyzed, i.e. growth of cells in hypoxia for 18, 72, and 96 hrs. (ZIP) [file pone.0187206.s004.zip › 72h/15)pfkfb all 72.jpg]

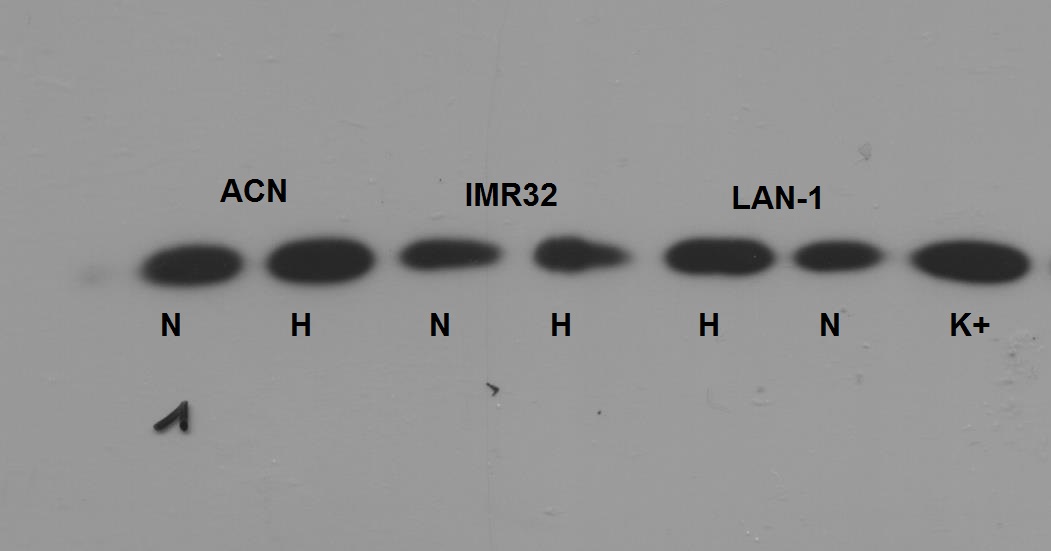

Supplement: S2 File — Original unadjusted Western blot films, subdivided in three folders corresponding to each of the hypoxic conditions analyzed, i.e. growth of cells in hypoxia for 18, 72, and 96 hrs. (ZIP) [file pone.0187206.s004.zip › 72h/17)vegf all 72.jpg]

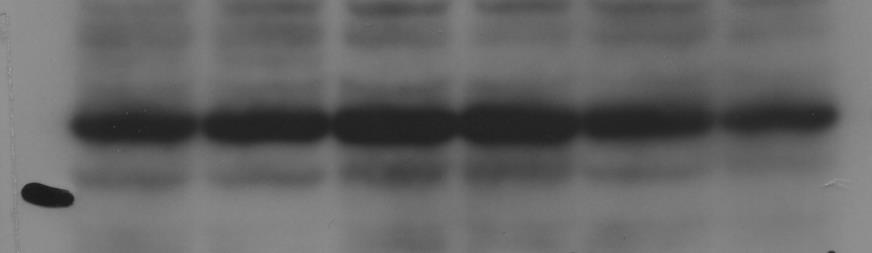

Supplement: S2 File — Original unadjusted Western blot films, subdivided in three folders corresponding to each of the hypoxic conditions analyzed, i.e. growth of cells in hypoxia for 18, 72, and 96 hrs. (ZIP) [file pone.0187206.s004.zip › 72h/18)actin all 72.jpeg]

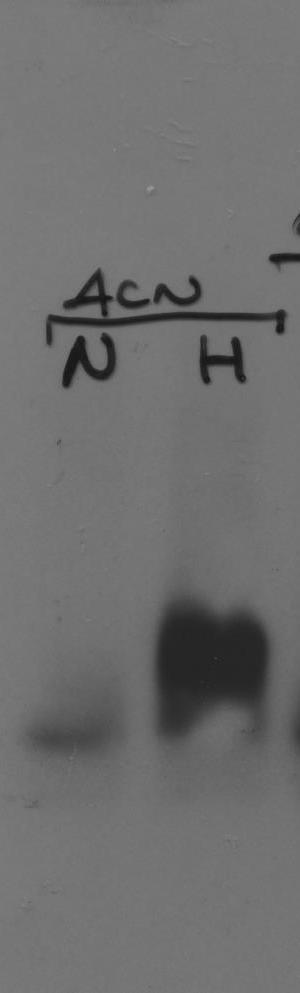

Supplement: S2 File — Original unadjusted Western blot films, subdivided in three folders corresponding to each of the hypoxic conditions analyzed, i.e. growth of cells in hypoxia for 18, 72, and 96 hrs. (ZIP) [file pone.0187206.s004.zip › 96h/04)hif ACN 96h.jpeg]

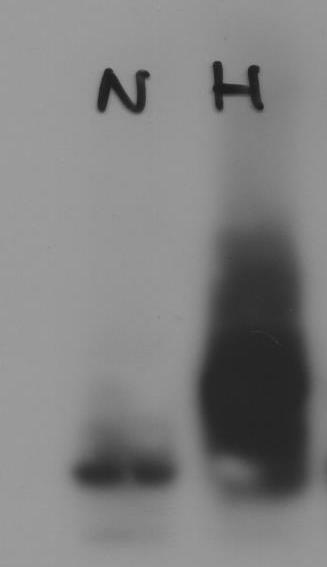

Supplement: S2 File — Original unadjusted Western blot films, subdivided in three folders corresponding to each of the hypoxic conditions analyzed, i.e. growth of cells in hypoxia for 18, 72, and 96 hrs. (ZIP) [file pone.0187206.s004.zip › 96h/05)hif IMR 96.jpeg]

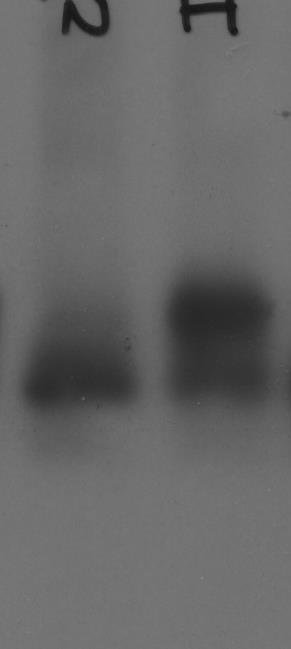

Supplement: S2 File — Original unadjusted Western blot films, subdivided in three folders corresponding to each of the hypoxic conditions analyzed, i.e. growth of cells in hypoxia for 18, 72, and 96 hrs. (ZIP) [file pone.0187206.s004.zip › 96h/06)hif LAN 96h.jpeg]

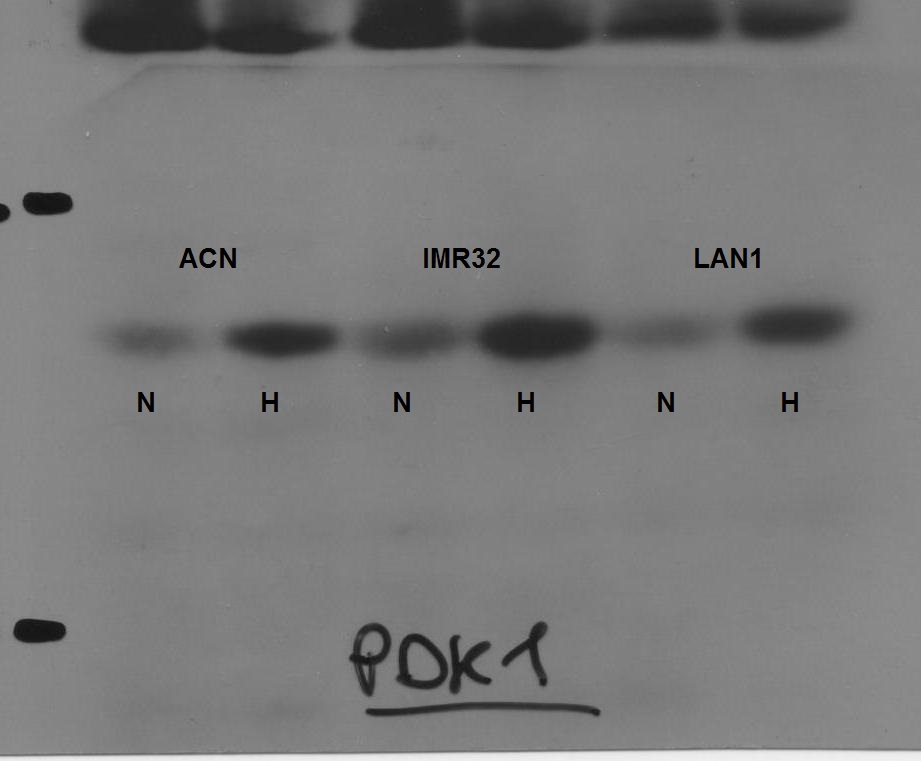

Supplement: S2 File — Original unadjusted Western blot films, subdivided in three folders corresponding to each of the hypoxic conditions analyzed, i.e. growth of cells in hypoxia for 18, 72, and 96 hrs. (ZIP) [file pone.0187206.s004.zip › 96h/10)pdk all 96.jpeg]

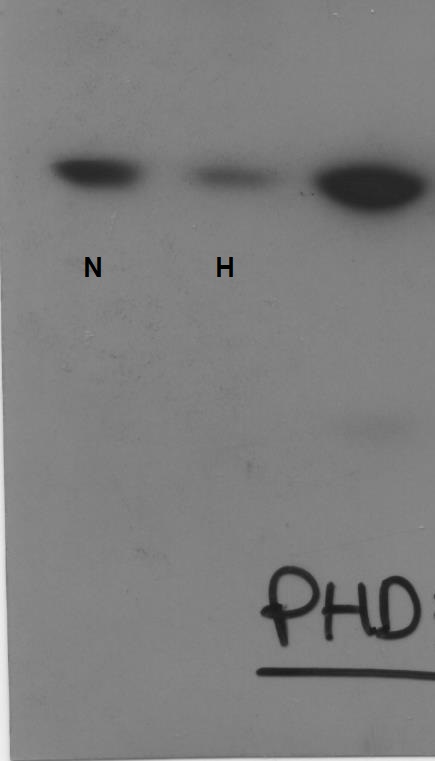

Supplement: S2 File — Original unadjusted Western blot films, subdivided in three folders corresponding to each of the hypoxic conditions analyzed, i.e. growth of cells in hypoxia for 18, 72, and 96 hrs. (ZIP) [file pone.0187206.s004.zip › 96h/13)phd3 ACN 96.jpeg]

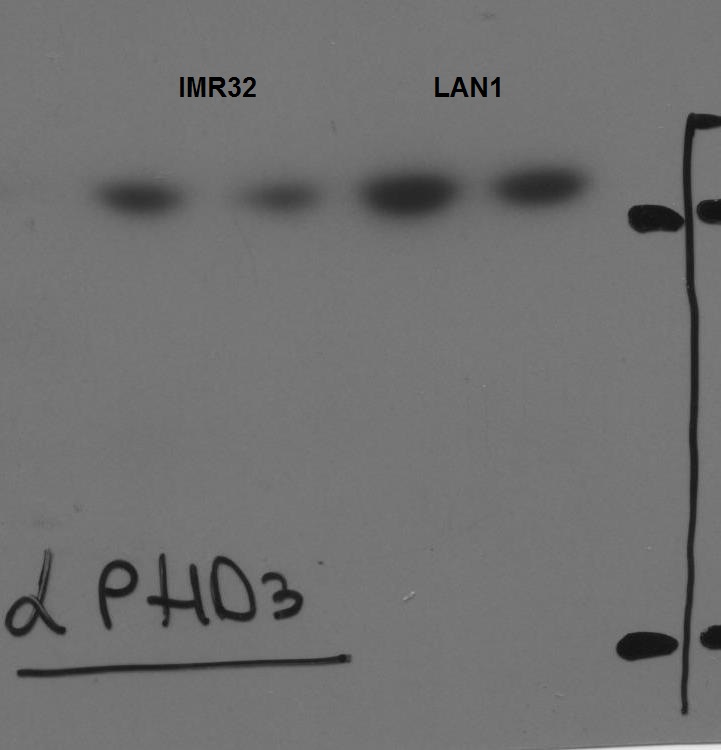

Supplement: S2 File — Original unadjusted Western blot films, subdivided in three folders corresponding to each of the hypoxic conditions analyzed, i.e. growth of cells in hypoxia for 18, 72, and 96 hrs. (ZIP) [file pone.0187206.s004.zip › 96h/14)phd3 IMR-LAN 96.jpeg]

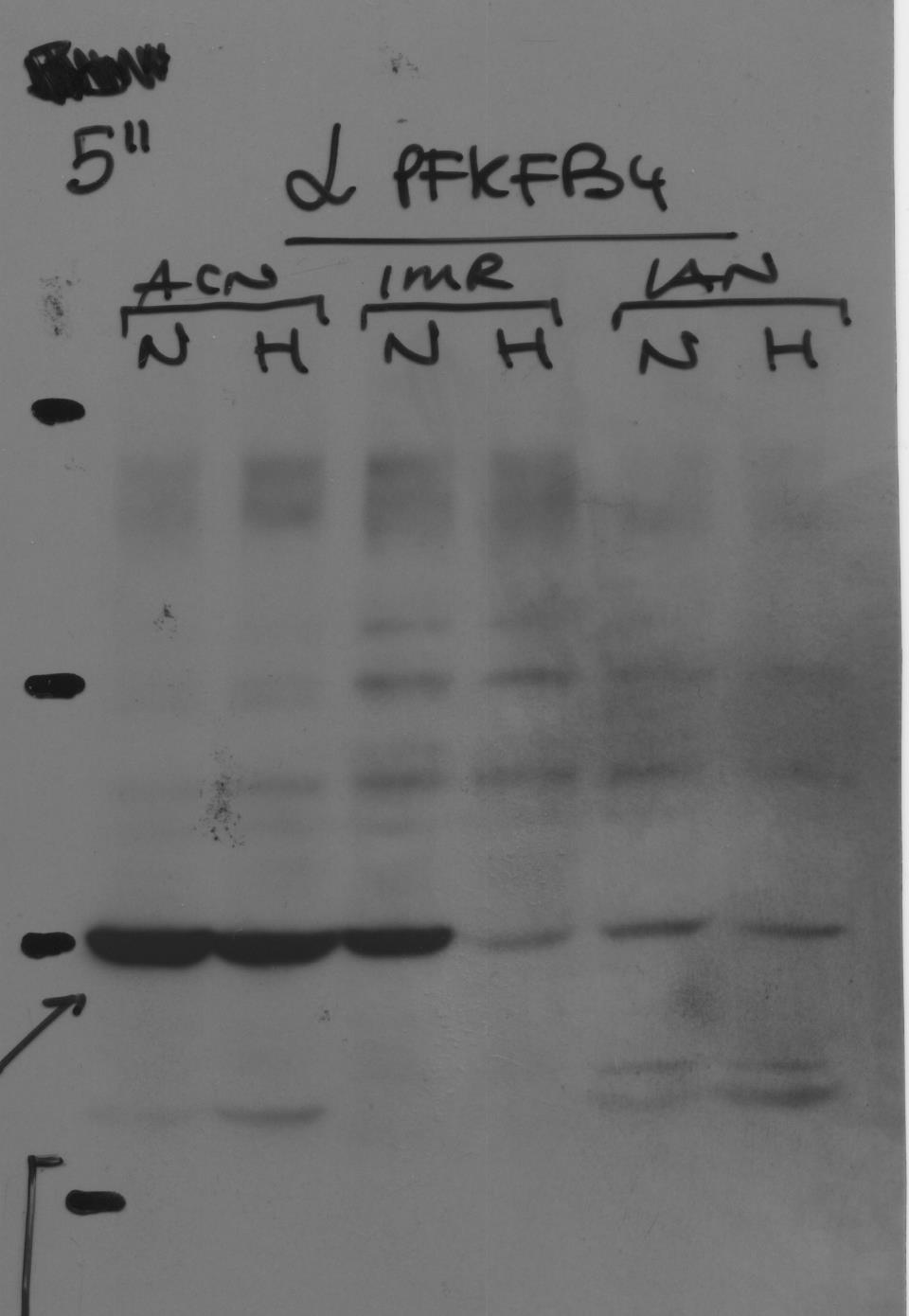

Supplement: S2 File — Original unadjusted Western blot films, subdivided in three folders corresponding to each of the hypoxic conditions analyzed, i.e. growth of cells in hypoxia for 18, 72, and 96 hrs. (ZIP) [file pone.0187206.s004.zip › 96h/16)pfkf all 96.jpg]

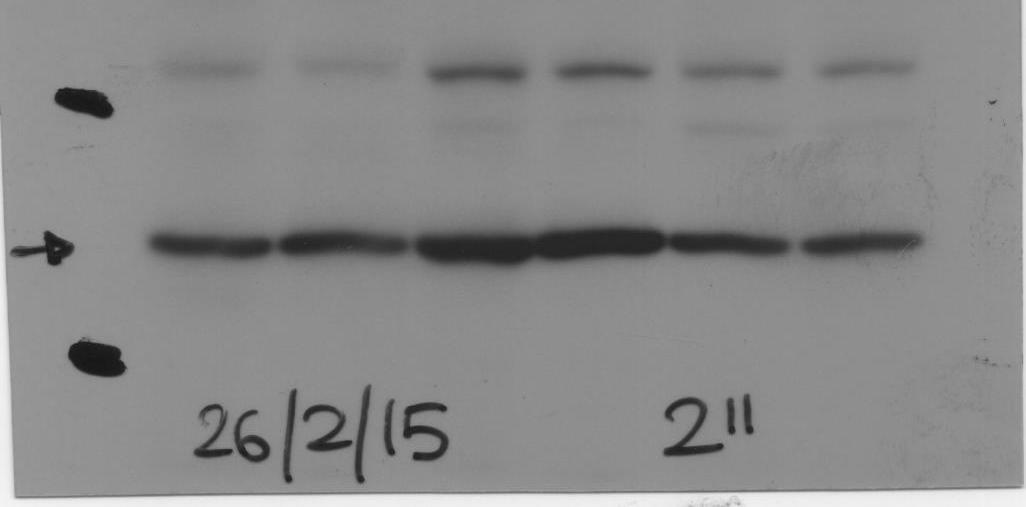

Supplement: S2 File — Original unadjusted Western blot films, subdivided in three folders corresponding to each of the hypoxic conditions analyzed, i.e. growth of cells in hypoxia for 18, 72, and 96 hrs. (ZIP) [file pone.0187206.s004.zip › 96h/19)actin all 96.jpeg]

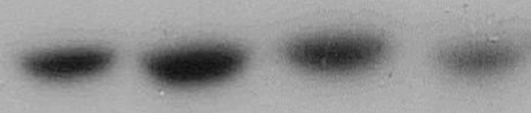

Supplement: S2 File — Original unadjusted Western blot films, subdivided in three folders corresponding to each of the hypoxic conditions analyzed, i.e. growth of cells in hypoxia for 18, 72, and 96 hrs. (ZIP) [file pone.0187206.s004.zip › 96h/20)vegf ACN-IMR 96.jpg]

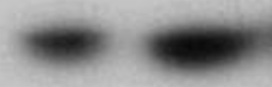

Supplement: S2 File — Original unadjusted Western blot films, subdivided in three folders corresponding to each of the hypoxic conditions analyzed, i.e. growth of cells in hypoxia for 18, 72, and 96 hrs. (ZIP) [file pone.0187206.s004.zip › 96h/21)vegf LAN 96.jpg]
